# Immunoblot Criteria for Diagnosis of Lyme Disease: A Comparison of CDC Criteria to Alternative Interpretive Approaches

**DOI:** 10.3390/pathogens12111282

**Published:** 2023-10-26

**Authors:** Richard Porwancher, Andrew Levin, Rosalie Trevejo

**Affiliations:** 1 Section of Allergy, Immunology, and Infectious Diseases, Department of Medicine, Rutgers Robert Wood Johnson Medical School, New Brunswick, NJ 08901, USA; 2 Princeton Infectious Diseases Associates, LLC, Plainsboro, NJ 08536, USA; 3 Kephera Diagnostics, LLC, Framingham, MA 01702, USA; alevin@kephera.com; 4 Epidemiologist, Acute and Communicable Disease Prevention, Oregon Health Authority, Portland, OR 97232, USA; rosalie.trevejo2@oha.oregon.gov

**Keywords:** Lyme disease, borreliosis, Western blot, immunoblot, line blot, interpretive criteria, alternative criteria, *Borrelia burgdorferi*, modified two-tier, two-tiered, single-tier

## Abstract

The current Centers for Disease Control and Prevention (CDC) interpretive criteria for serodiagnosis of Lyme disease (LD) involve a two-tiered approach, consisting of a first-tier EIA, IFA, or chemiluminescent assay, followed by confirmation of positive or equivocal results by either immunoblot or a second-tier EIA. To increase overall sensitivity, single-tier alternative immunoblot assays have been proposed, often utilizing antigens from multiple *Borrelia burgdorferi* strains or genospecies in a single immunoblot; including OspA and OspB in their antigen panel; requiring fewer positive bands than permitted by current CDC criteria; and reporting equivocal results. Published reports concerning alternative immunoblot assays have used relatively small numbers of LD patients and controls to evaluate novel multi-antigen assays and interpretive criteria. We compared the two most commonly used alternative immunoblot interpretive criteria (labeled A and B) to CDC criteria using data from multiple FDA-cleared IgG and IgM immunoblot test kits. These single-tier alternative interpretive criteria, applied to both IgG and IgM immunoblots, demonstrated significantly more false-positive or equivocal results in healthy controls than two-tiered CDC criteria (12.4% and 35.0% for Criteria A and B, respectively, versus 1.0% for CDC criteria). Due to limited standardization and high false-positive rates, the presently evaluated single-tier alternative immunoblot interpretive criteria appear inferior to CDC two-tiered criteria.

## 1. Introduction

CDC serologic approaches to LD diagnosis currently utilize a two-tiered method: serum from patients with clinically suspected LD are first tested by EIA, IFA, or chemiluminescent assay, followed by a second-tier EIA or immunoblot assay to confirm positive or equivocal first-tier results [1]. First-tier EIAs and chemiluminescent assays for polyvalent IgG/IgM antibodies to *B. burgdorferi* may yield false-positive rates between 2% and 5% when using recombinant proteins [2,3] and between 10% and 15% when using whole-cell lysate [4,5]. Because of concerns about false-positive first-tier test results, particularly in low-risk settings, the CDC recommends a confirmatory second-tier test [1].

The most common manifestation of early Lyme disease is erythema migrans, a skin lesion observed in 70% to 90% of cases [6,7]. Because serologic testing is insensitive for early Lyme disease, patients presenting with erythema migrans are best diagnosed on clinical grounds [6,8]. Experienced physicians practicing in endemic communities can correctly diagnose erythema migrans in 72% to 92% of cases [9]. There is general agreement that patients with suspected extra-cutaneous Lyme disease, such as Lyme arthritis, carditis, and neuroborreliosis, should have serologic testing [10,11]. Most testing in the US, however, is performed in a setting of low pretest risk (<5%), with overall estimates ranging from 0.6% to 12.0% [12,13,14,15]. A recent report by Kobayashi et al. [16] from a university referral clinic noted that physician misinterpretation of serologic tests, particularly IgM immunoblots, played a significant role in misdiagnosis. Conant et al. [14] observed that 42% of primary care providers practicing in a high-incidence state attributed chronic neurocognitive symptoms to Lyme disease based solely on positive IgM immunoblot test results, a departure from current guidelines [10,17]. The above reports are consistent with prior studies and illustrate the potential for harm associated with false-positive Lyme disease serology [18,19,20].

Between 1995 and 2019, immunoblotting was the sole serologic technique advised by the CDC to confirm positive or equivocal results from first-tier screening assays [1]; thus, immunoblotting has been a critical tool for LD serodiagnosis. There are two principal formats for immunoblotting: Western blots and line blots. Western blots involve electrophoretic separation of *B. burgdorferi* proteins (typically whole-cell lysate) using a polyacrylamide gel, followed by transfer (blotting) of these proteins onto nitrocellulose membrane strips; after incubating the strips with patient serum, IgG or IgM antibodies are detected by EIA and band density compared to a weak-positive control [21,22]. Because traditional Western blots utilize whole-cell lysates, non-specific proteins may co-migrate with CDC-advocated antigens, reducing band specificity [23]. Bands corresponding to specific *B. burgdorferi* proteins are routinely identified using monoclonal antibodies on control strips for FDA-cleared kits; the latter technique helps distinguish p30 (periplasmic substrate-binding protein) from p31 (OspA), as well as proteins with variable electrophoretic migration, such as p23 (OspC with migration ranging from 21- to 25-kDa) and p93 (a protoplasmic cylinder antigen with migration ranging from 83- to 100-kDa) [21]. In contrast, line blots utilize either purified or recombinant *B. burgdorferi* proteins imprinted in a linear fashion onto nitrocellulose membrane strips; incubation with patient serum and EIA detection of IgG or IgM antibodies are accomplished as with Western blots [24]. By utilizing purified or recombinant antigens, line blots eliminate the potential for cross-reactions due to co-migrating proteins. Goettner et al. [24] also observed improved sensitivity for European neuroborreliosis using recombinant-based line blots, although they employed antigens from multiple European *B. burgdorferi* genospecies. For either technique, immunoblot band density can be compared to a weak-positive control band by visual examination or spectrophotometer; each band is reported as positive (density greater than or equal to the weak-positive band) or negative [25]. Assay standardization requires reproducibility studies that include justification of the choice of weak-positive band density [10,21].

CDC interpretive criteria were chosen to maintain high specificity as second-tier assays, typically 97% to 99% for combined IgG and IgM immunoblots [5,26]. CDC criteria for positive IgM immunoblots [27] require the presence of at least two of the following three bands: 23-, 39-, and 41-kDa. CDC criteria for positive IgG immunoblots [27] require at least five of the following ten bands: 18-, 23-, 28-, 30-, 39-, 41-, 45-, 58-, 66-, and 93-kDa; only IgG immunoblots are advised for diagnosis more than 30 days after disease onset, although some investigators have suggested using IgM immunoblots to diagnose Lyme neuroborreliosis up to 6 weeks after disease onset [10].

Due to low sensitivity of the CDC two-tiered approach using immunoblots for diagnosis of early LD [8,10], alternative approaches have been proposed. A modified two-tiered (MTT) all-EIA assay was recently FDA-cleared and is considered an acceptable alternative to the standard CDC two-tiered method [1,3]. MTT approaches may demonstrate a higher sensitivity for early LD than two-tiered methods using immunoblots (67–74% versus 41–50%, respectively), but without significant loss of specificity (98–99% versus 96–100%, respectively) [2,3,5]. Some MTT approaches, however, do not routinely identify which *B. burgdorferi* antigens are responsible for a positive result. When potentially cross-reacting medical conditions are suspected, such as syphilis, tick-borne coinfections (e.g., human granulocytic anaplasmosis), and some viral illnesses (e.g., Epstein–Barr virus), greater diagnostic information about the immune response to *B. burgdorferi* may be afforded by IgG immunoblots [4,10].

To improve sensitivity, immunoblots have been utilized by some investigators and CLIA-certified laboratories as single-tier alternatives to the CDC two-tiered paradigm ([28,29,30,31], Supplementary File S1, Laboratories A and B [32,33]). See Table 1 and Table 2 for alternative interpretive Criteria A and B, respectively; no confirmatory assay is required for results positive by these immunoblot criteria. Additional bands at the 31- and 34-kDa positions, representing antibodies to OspA and OspB antigens, respectively, were utilized and interpretive criteria liberalized to increase sensitivity. Limited data exist, however, concerning the contributions of the 31- and 34-kDa bands toward diagnosis. Previous studies suggest that antibodies to OspA and OspB are more commonly seen in late-stage LD than early disease [8,34,35]. Hilton et al. [36] reported 8% improved sensitivity over CDC two-tiered criteria by including these antigens in the overall immunoblot panel, principally in patients with partially treated late-stage LD; in contrast, Trevejo et al. [8] reported only a 0.8% improvement in detecting early LD by including the 31- and 34-kDa bands in the CDC immunoblot panel. Patients who previously received an OspA-based LD vaccine would be expected to demonstrate false-positive reactions to the 31-kDa band. Although the OspA-based vaccine has not been marketed since 2002 [21], newer OspA-based vaccines are currently in development [37].

Studies of analytical performance are routinely evaluated by CLIA-certified laboratories for in-house tests (also called laboratory-developed tests). Although independent verification of laboratory-developed test performance is not required for CLIA-certified laboratories, studies of in-house analytical performance and proficiency testing are not equivalent to the clinical accuracy studies required for FDA clearance (e.g., 510(k) application) [38]. Alternative Criteria A, as utilized by Laboratory A, were based on studies by Shah et al. [29,31] and Liu et al. [30], which employed antigens from multiple different *B. burgdorferi* strains and/or genospecies in either a Western blot or line blot format. European LD can be caused by multiple different genospecies of *B. burgdorferi* sensu lato, including *B. burgdorferi* sensu stricto, *B. garinii*, and *B. afzelii*. Except for isolated cases of *B. mayonii* reported from the upper Midwestern US [39], North American LD is caused nearly exclusively by *B. burgdorferi* sensu stricto [10]; current FDA-cleared immunoblots utilize the B31 strain of this genospecies.

Shah et al. [29] employed both the B31 and 297 strains of *B. burgdorferi* sensu stricto for IgG and IgM Western blots for the diagnosis of US Lyme disease. Employing 35 US LD sera from the CDC, Shah et al. [29] determined that the sensitivity of their single-tier two-strain Western blot, when positive by Criteria A, was 20% greater than the combination of single-tier MarDx Marblot IgG and IgM Western blots interpreted using the CDC criteria (MarDx Diagnostics, Inc., Carlsbad, CA, USA) (34/35 sera (97%) vs. 27/35 sera (77%), respectively, *p* ≤ 0.046 by two-tailed McNemar’s test). Three possible explanations for this difference in sensitivity between laboratories are: (i) fewer bands were required to consider an immunoblot positive, (ii) weak-positive bands were interpreted differently, and (iii) multiple *B. burgdorferi* strains were employed for antibody detection. Shah et al. [29] observed a 3% gain in overall sensitivity when MarDx Marblot Western blots performed by the CDC were reinterpreted as positive using Criteria A, an additional 14% improvement when both IgG and IgM MarDx Marblots were rerun in-house and interpreted using Criteria A, and an additional 3% gain in sensitivity when immunoblots utilized whole-cell lysate from two *B. burgdorferi* strains; the above observations suggest that the difference in Western blot sensitivity between laboratories was primarily related to band interpretation.

Shah et al. [29] also compared single-tier Criteria A to single-tier CDC criteria in 276 controls using their two-strain Western blot (86 sera from healthy controls and 190 sera from patients with potentially cross-reacting conditions); this study documented a significant loss of specificity using Criteria A versus CDC criteria, principally in patients with potentially cross-reacting conditions (247/276 (89.5%) vs. 268/276 (97.1%), *p* ≤ 0.001 by two-tailed McNemar’s test). Shah et al. [29] proposed using a laboratory-developed immunoblot employing recombinant OspA to clarify specimens demonstrating positive 31-kDa bands; eliminating 31-kDa positive controls not confirmed by the recombinant OspA immunoblotting improved overall specificity to 263/276 (95.3%).

Even though North American LD is caused nearly exclusively by *B. burgdorferi* sensu stricto, a line blot proposed by Liu et al. [30] for US diagnosis utilized recombinant antigens from 8 different European and US strains and/or genospecies of *B. burgdorferi*, again interpreted using Criteria A; using a single-tier approach, investigators reported 100% sensitivity among 26 U.S. LD sera and 97% specificity among 152 control sera. These 152 controls included 116 samples with potentially cross-reacting medical conditions and 36 undefined control sera (11 from proficiency tests and 25 samples from the CDC). The above study samples were also tested by Western blot (included 17 LD sera and 25 controls tested at the CDC using an unspecified Western blot kit, as well as 9 LD sera and 127 controls tested using the two-strain Western blot described above [29]). When the above Western blots were interpreted using CDC criteria as single-tier assays, there was no significant difference in sensitivity or specificity compared to the proposed single-tier line blots interpreted using Criteria A. A total of 22/26 U.S. LD sera (85%) were positive by Western blot versus 26/26 LD sera (100%) positive by the proposed line blot (*p* = 0.13 by two-tailed McNemar’s test). The specificity of single-tier Western blotting using CDC criteria was 98% (149/152) versus 97% (148/152) for the single-tier line blot using Criteria A.

Additional methodological issues complicate interpretation of both the Shah [29] and Liu [30] studies: since a portion of the control sera from both studies included specimens from laboratory proficiency tests, these samples may include pooled sera or duplicates and may not be comparable to studies where each control specimen corresponds to a separate individual. As many as 71 healthy controls studied by Shah et al. [29,31] and all 152 controls evaluated by Liu et al. [30] appeared to be pre-screened for antibodies to Lyme disease before testing using alternative immunoblot techniques; these control samples may not demonstrate the same immunoblot results as unscreened controls. Finally, justification of weak-positive band density and immunoblot reproducibility studies were not reported by either Shah [29,31] or Liu [30].

CLIA-certified laboratories employing alternative immunoblot techniques and interpretive criteria differ in reporting and resolution of equivocal immunoblot results (Table 1 and Table 2). Criteria A report equivocal results for laboratory-developed Western blots but not line blots; recommendations from Laboratory A for resolution of equivocal Western blots include either repeating the immunoblot in 6 to 8 weeks or employing a second assay (e.g., a recombinant OspA-based laboratory-developed immunoblot [29,32]). Criteria B, adapted from Tilton et al. [28], utilize the same interpretive criteria for Western blots and line blots and consider the presence of either one or two designated IgG bands or one designated IgM band as equivocal results [33]. Although the Tilton study [28] does not address resolution of equivocal immunoblots, repeating the immunoblot in 2 to 4 weeks has been recommended by Laboratory B (Supplementary File S1) [33]; for patients with chronic illnesses, changes in serologic results might not occur, leaving the clinician without a standardized means to resolve equivocal findings. The CDC reports immunoblots as either positive or negative and utilizes the same interpretive criteria for Western blots and line blots; the latter approach avoids equivocal immunoblot results and permits using immunoblots to resolve equivocal first-tier assays.

A small study by Fallon et al. [40] compared laboratory-developed immunoblot assay results from two “Lyme specialty” laboratories with results obtained using CDC-advocated techniques at one university-based reference laboratory and one commercial laboratory. Samples from 37 US patients with post-treatment LD syndrome and 40 healthy controls were processed in a blinded fashion at all 4 laboratories. Only positive immunoblot results were compared to CDC criteria. The Fallon study [40] raised concerns about false-positive immunoblot results for IgG and IgM antibodies at one “Lyme specialty” laboratory employing a multi-strain Western blot interpreted using alternative criteria; 57.5% of healthy controls demonstrated falsely positive IgG or IgM immunoblots at this specialty laboratory. Investigators demonstrated improved specificity by utilizing a second-tier FDA-cleared EIA to supplement IgG immunoblot results positive by alternative criteria [40]. The Fallon study [40] failed, however, to evaluate the combined sensitivity of IgG and IgM immunoblots, address the management of equivocal immunoblot results, or test patients with potentially cross-reacting medical conditions. In contrast to CDC guidelines [27], Fallon also permitted use of IgM immunoblotting alone for diagnosis of late-stage Lyme disease.

Since the Fallon study [40] was published in 2014, the interpretive criteria for laboratory-developed immunoblots have changed, dropping the use of the 20- and 35-kDa bands [30,32,33]; also, line blots utilizing recombinant antigens from multiple *B. burgdorferi* genospecies have been introduced [30]. The purpose of the current study is to compare the performance of updated alternative immunoblot criteria, herein labeled Criteria A and B, to CDC criteria utilizing previously unpublished data from three FDA-cleared immunoblot test kits (two Western blot kits and one line blot kit). Unlike the Fallon study [40], we assess the combined sensitivity of IgG and IgM immunoblots for early LD, evaluate the impact of equivocal immunoblot results on overall performance, assess immunoblot specificity among patients with potentially cross-reacting medical conditions, and, when applying CDC two-tiered immunoblot criteria, utilize only IgG antibody for diagnosis of LD when the duration of illness exceeds 30 days. Since MTT approaches to serodiagnosis have been FDA-cleared since 2019 [10], we also compare the performance of MTT assays to alternative immunoblot criteria.

## 2. Methods

### 2.1. Dataset Descriptions

Eight different datasets were employed to assess the performance of six different FDA-cleared immunoblot test kits for antibodies against the B31 strain of *Borrelia burgdorferi*, including three Western blot test kits and three line blot test kits (see Table 3 and Supplementary File S2 for full descriptions). These immunoblot test kits were chosen based on utilization in CDC-sponsored research, availability of individual results for the 31- and 34-kDa bands, and/or recent FDA-clearance, and are summarized below. Three datasets (Immunetics QualiCode Western blots (510(k), dataset 1), MarDx Marblot Western blots (Trevejo [8], dataset 4), and the Viramed ViraStripe line blots from the CDC Lyme Serum Repository (LSR) (Molins [5], dataset 8)) included sufficient individual sample test results to assess both CDC and alternative immunoblot interpretive criteria, but only datasets 1 and 4 reported 31- and 34-kDa band results. An additional five datasets were evaluated to assess the range of immunoblot performance using CDC criteria among kits and across different datasets using the same kit. Immunoblots were performed on all samples, regardless of other assay results (e.g., EIA) or the duration of infection. All immunoblot band results were visually interpreted. Although spectrophotometric readers were available for the EUROIMMUN IgG and IgM Western blots (510(k), dataset 2) and the Viramed ViraStripe IgG and IgM line blots (Molins [5], (LSR, dataset 8)), incorporating the latter methodology would have complicated assay comparisons [5,41].

Data on separate, single-tier IgG and IgM immunoblot test kit performance, interpreted using CDC criteria [27], were available for all datasets except dataset 5 (the MarDx Marblot Western blot (Johnson [26]) dataset). Although immunoblots are FDA-cleared for confirmation of first-tier LD assays, test manufacturers provide single-tier sensitivity and specificity data in their 510(k) FDA applications and package inserts. Individual band frequencies in IgG and IgM immunoblots from healthy controls from endemic and non-endemic communities are listed in Supplementary Tables S1 and S2, respectively, for heuristic comparisons. Supplementary Table S3 (Section A) reports immunoblot band frequencies among patients with potentially cross-reacting medical conditions from the LSR using both Viramed ViraStripe line blots and MarDx Marblot Western blots (Molins [5], dataset 8). Supplementary Table S3 (Section B) reports IgG immunoblot FPRs in different datasets based on the number of CDC-advocated IgG bands utilized to consider a test positive; these data explore the impact of utilizing fewer than five bands as a cutoff.

Combined IgG and IgM immunoblot performance data, interpreted using both single-tier and two-tiered CDC criteria, were available for MarDx Marblot Western blots (Trevejo [8], dataset 4), MarDx Marblot Western blots (Johnson [26], dataset 5), and the Viramed ViraStripe line blots (Molins [5], (LSR, dataset 8)). When applying two-tiered CDC criteria, only IgG immunoblots were utilized for LD diagnosis when the duration of illness exceeded 30 days. MTT all-EIA performance using both IgG and IgM antibodies was available only for the Viramed ViraStripe line blots (Molins [5], (LSR, dataset 8)) (see Supplementary File S2).

In addition to the standard 10 CDC-advocated IgG bands, only the Immunetics QualiCode Western blots (510(k), dataset 1) and MarDx Marblot Western blots (Trevejo [8], dataset 4) reported IgG antibody results to the 31- and 34-kDa bands in healthy controls, permitting a full evaluation of IgG immunoblot specificity using alternative interpretive Criteria A and B. Only dataset 4 (MarDx Marblot Western blots (Trevejo [8])) reported the 31- and 34-kDa band results for both IgG and IgM Western blots. Separate analyses of alternative interpretive criteria, both with and without the 31- and 34-kDa bands, were performed on dataset 4 to evaluate the impact of these bands on assay performance.

The remaining five datasets (EUROIMMUN IgG and IgM Western blots (510(k), dataset 2), MarDx Marblot Western blots (510(k), dataset 3), Trinity Biotech MarStripe line blots (510(k), dataset 6), the Gold Standard Diagnostics line blots (510(k), dataset 7), and the MarDx Marblot Western blots from Molins et al. [5], (LSR, dataset 8)) reported summaries of separate IgG and IgM immunoblot performance using CDC criteria without the 31- or 34-kDa bands.

Separate immunoblot results for healthy controls from both endemic and non-endemic areas were available for all datasets except the MarDx Marblot Western blots (Trevejo [8], dataset 4), the MarDx Marblot Western blots (510(k), dataset 3), and the Trinity Biotech MarStripe line blots (510(k), dataset 6); dataset 4 utilized healthy controls from only endemic communities, while the latter two datasets (3 and 6) utilized healthy controls from both endemic and non-endemic communities. The healthy controls from datasets 3 and 6 potentially overlap because they were both used for 510(k) FDA applications by the same manufacturer (Trinity Biotech). Statistical comparisons of specificity utilized data from all healthy controls from each dataset (i.e., included sera from healthy individuals living in either endemic or non-endemic areas). Immunoblot results from patients with potentially cross-reacting medical conditions were available only for CDC criteria from the Immunetics QualiCode Western blots (510(k), dataset 1). In contrast, the Viramed ViraStripe line blots (Molins [5], (LSR, dataset 8)) included data for all interpretive criteria in individuals with potentially cross-reacting conditions. Neither dataset 1 nor 8 recorded 31- and 34-kDa band results for this subset of controls. Specificity comparisons in patients with potentially cross-reacting conditions were therefore limited to samples from the CDC LSR (dataset 8).

The use of additional immunoassays varied by dataset (see Supplementary File S2). Two different MTT assays were assessed using the CDC Lyme Serum Repository (Molins [5], (LSR, dataset 8)), as summarized below. One MTT utilized a first-tier whole-cell EIA to detect polyvalent IgG and IgM antibodies to *B. burgdorferi*; positive or equivocal first-tier results were confirmed by a second-tier EIA for polyvalent IgG and IgM antibodies to the C6 peptide. The second MTT utilized a first-tier EIA for polyvalent IgG and IgM antibodies to a combination of VlsE1 and pepC10 antigens; positive or equivocal first-tier results were confirmed by monovalent whole-cell EIAs for IgG and IgM antibodies to *B. burgdorferi*. Both MTT assays required positive or equivocal results for both tiers to consider the overall assay positive.

Because serodiagnosis of early LD is the most challenging [10], assay sensitivity was determined using sera obtained within 90 days of disease onset for all datasets; results are reported on a per sample rather than per patient basis. The sensitivity of alternative immunoblot interpretive criteria for later-stage LD was evaluated using the Viramed ViraStripe line blots (Molins [5], (LSR, dataset 8)), but this dataset did not include the 31- and 34-kDa bands.

Serologic results for individual serum specimens from the Immunetics QualiCode Western blots (510(k), dataset 1) and the MarDx Marblot Western blots (Trevejo [8], dataset 4) are available in Supplementary Files S3 and S4, respectively. The Viramed ViraStripe line blots (Molins [5], (LSR, dataset 8)) used partially blinded serologic data from the CDC Lyme Serum Repository that were provided to one of the authors (R.P.) under a material transfer agreement.

### 2.2. Statistical Analyses

Statistical analyses were performed using MedCalc software, version 20.216 (2023) (MedCalc Software Ltd., Ostend, Belgium), except as indicated below. Two-sided confidence intervals for proportions were calculated using the Newcombe–Wilson method without continuity correction (α = 0.05) [42]. McNemar’s test was utilized to compare proportions from paired data from a single study (two-tailed α = 0.05); in case of minimal missing data for paired comparisons, then the least extreme difference in data distribution was used to calculate *p*-values and confidence intervals using McNemar’s test (representing the minimum difference in paired assay results). Fisher’s exact test was used to compare proportions from independent datasets or when individual results for paired data were unavailable (two-tailed α = 0.05). In case of multiple comparisons within the same dataset, a Bonferroni correction was applied to limit false discovery: any individual *p*-value was considered significant only if the cumulative *p*-value for all comparisons was ≤ 0.05. Positive and negative likelihood ratio were generated for all criteria in each dataset. A positive likelihood ratio was defined as: $\frac{sensitivity}{1-specificity\;}$. A negative likelihood ratio was defined as: $\frac{1-sensitivity\;}{specificity}$. Two-sided confidence intervals for likelihood ratios associated with a given criterion for a given dataset were calculated using the method of Simel et al. (α = 0.05) [43].

Only non-overlapping datasets were used for meta-analyses. If either Lyme disease patients or controls from two datasets overlapped, then the larger of the two datasets was used for meta-analyses. Fixed effect meta-analyses of sensitivities and FPRs were performed when assessing separate studies using a single immunoblot kit, while random effect meta-analyses were performed when assessing separate studies using multiple different immunoblot kits. When evaluating paired data from a given study, the differences in FPRs or sensitivities for different interpretive criteria were expressed as a proportion of either the control population (for FPRs) or the diseased population (for sensitivity) for that study. When evaluating paired data from multiple separate studies, a random effect meta-analysis of these differences, expressed as proportions, was used to generate a composite difference in either the FPR or the sensitivity between interpretive criteria, as well as 95% confidence intervals. The above approach utilizes a Freeman–Tukey transformation [44] to calculate the weighted summary proportion under the random effect model of DerSimonian and Laird [45]. Data heterogeneity was assessed using the I^2^ statistic.

Point estimates of positive and negative likelihood ratios were derived from meta-analytic composite sensitivity and FPR of each criterion and used for heuristic comparisons as advised by Trikalinos et al. [46]. Comparing immunoassay diagnostic performance through likelihood ratios also utilized guidance provided by Biggerstaff [47], wherein an assay with higher positive and lower negative likelihood ratios than a competing assay is considered superior, regardless of the pretest probability of disease.

We also utilized test accuracy to choose between two diagnostic tests with different performance characteristics. When Test A demonstrates higher sensitivity but lower specificity than Test B, then Equation (1) can be used to calculate the pretest probability of LD where test accuracy is equivalent [47]:

(1) $$Pretest\;probability={\left[1+\frac{\left(Sensitivity\;A-Sensitivity\;B\right)}{\left(Specificity\;B-Specificity\;A\right)}\right]}^{-1}$$

Based on the pretest probability of LD in a given clinical setting, healthcare providers can choose the more accurate of the two tests. Comparisons of test accuracy between alternative immunoblot criteria and two-tiered CDC criteria were explored using the Viramed ViraStripe line blot (LSR) dataset in Section 3.6.

We also assumed that if a given criterion could demonstrate greater than 50% accuracy in a given clinical setting, then that criterion could be utilized for clinical decision-making; the latter goal is mathematically equivalent to demonstrating a positive predictive value >50%. Utilizing the positive likelihood ratio associated with a given diagnostic criterion for a given dataset, the pretest probability of LD required for that criterion to demonstrate a positive predictive value (PPV) ≥50% was calculated using Equation (2) below:

(2) $$Pretest\;probability={\left(1+positive\;likelihood\;ratio\right)}^{-1}$$

Equation (2) was derived from Bayes theorem, expressed in odds ratio format, by assuming that the post-test probability of LD is 50% (i.e., post-test odds = 1) for a test positive by a given criterion and solving for the pretest probability of LD [48].

Applying Equation (2) to the Viramed ViraStripe line blot (Molins [5], (LSR, dataset 8)), we determined the pretest probability of Lyme disease required for each criterion to demonstrate a PPV ≥ 50% (Section 3.6); the latter pretest probability represents a decision threshold for the clinical application of that criterion. Since the latter dataset did not measure antibodies to OspA and OspB, it is possible that we underestimated the sensitivity of alternative immunoblot criteria for that dataset; we therefore performed one-way sensitivity analyses using the upper bound of the 95% confidence intervals of the positive likelihood ratios of the most sensitive alternative criteria to predict test performance, had the dataset included the latter antibodies.

## 3. Results

### 3.1. Immunoblot Band Frequencies in Controls: Impact of Varying Cutoffs on Immunoblot Specificity Utilizing CDC-Advocated Bands

Data concerning immunoblot band frequencies in controls are necessary to choose interpretive cutoffs for disease categorization. Supplementary Tables S1 and S2 list individual band frequencies in controls from endemic and non-endemic communities, respectively, for all datasets except dataset 5, as well as FPRs using single-tier CDC interpretive criteria for both IgG and IgM immunoblots; separate IgG and IgM immunoblot results were unavailable for dataset 5. Heuristic comparisons demonstrated significant variations in band frequency and FPRs using CDC criteria among datasets, both among kits and among datasets using the same kit. In general, band frequencies and FPRs were higher in endemic than non-endemic communities. False-positive tests were more frequent among IgM immunoblots than IgG immunoblots. FPRs in healthy controls from each dataset (inclusive of both endemic and non-endemic control sera) ranged from 0.5% to 3.0% for IgG immunoblots and from 0% to 7.9% for IgM immunoblots when using a single-tier approach.

It is important to note that the composition of patient panels with potentially cross-reacting medical conditions varied widely among datasets (see Supplementary File S2), reducing their value for meta-analyses. We therefore limited comparative analyses of LD assays among patients with potentially cross-reacting conditions to samples from the CDC Lyme Serum Repository (Molins [5], (LSR, dataset 8)), the only dataset with sufficient individual immunoblot results to determine the performance of alternative criteria in this group (Section 3.6). Supplementary Table S3 (Section A) reports individual band frequencies and FPRs using single-tier CDC criteria for IgG and IgM immunoblots among LSR patients with potentially cross-reacting conditions (i.e., Viramed ViraStripe line blots and MarDx Marblot Western blots). Individual band frequencies and FPRs for IgM immunoblots listed in Table S3 (Section A) varied by kit but appeared heuristically higher than the band frequencies and FPRs of the same IgM immunoblot kits among healthy LSR non-endemic controls (Supplementary Table S2); differences in IgM FPRs between control groups were not statistically significant. IgG immunoblot band frequencies and FPRs listed in Table S3 (Section A) among controls with potentially cross-reacting conditions also varied by kit but appeared heuristically similar to IgG immunoblot results reported using the same kits in healthy non-endemic controls (Supplementary Table S2).

Because of data heterogeneity, random effect meta-analyses were needed to determine the impact of different cutoffs on immunoblot specificity. Supplementary Table S3 (Section B) reports single-tier IgG immunoblot specificity for three datasets (Viramed ViraStripe IgG line blot (LSR, dataset 8), Immunetics QualiCode IgG Western blot (510(k), dataset 1), and MarDx Marblot IgG Western blot (Trevejo [8], dataset 4)) if fewer than five of ten CDC-advocated IgG bands were sufficient to consider an immunoblot positive; only these three datasets contained sufficient individual immunoblot data to calculate FPRs using different band cutoffs. Using either three or four IgG bands to consider an immunoblot positive led to significantly higher FPRs than the standard five-band cutoff. Random effect meta-analyses demonstrated FPRs of 15.7% using a three-band cutoff (95% CI: 8.2% to 25.2%), 6.5% using a 4-band cutoff (95% CI: 4.1% to 9.4%), and 2.2% using a 5-band cutoff (95% CI: 1.3% to 3.4%). These data illustrate the risk of generating false-positive results via assigning diagnostic significance to fewer than 5 of 10 CDC-advocated IgG bands. When the 31- and 34-kDa bands were included in the immunoblot panel, FPRs were even higher using alternative cutoffs for the Immunetics QualiCode IgG Western blot (510(k)) dataset, but were unchanged for the MarDx Marblot IgG Western blot (Trevejo [8]) dataset (see Table S3 (Section B)).

For single-tier IgM immunoblots, requiring only one of three CDC-advocated IgM bands to consider an immunoblot positive would have led to significant loss of specificity compared to standard CDC criteria in healthy controls. Individual FPRs for IgM immunoblots using a one-band cutoff were 21.1% for the MarDx Marblot IgM Western blot (Trevejo [8]) dataset, and 24.1% for the Viramed ViraStripe IgM line blot (LSR) dataset; a random effect meta-analysis using healthy controls from these two datasets demonstrated a composite FPR of 23.9% (95% CI: 18.7% to 29.4%) using a one-band cutoff and 7.1% (95% CI: 3.8% to 11.3%) using a standard two-band cutoff. High IgM FPRs were observed using a one-band cutoff even without including controls with potentially cross-reacting medical conditions.

### 3.2. Specificity of Single-Tier IgG Immunoblots That Include the 31- and 34-kDa Bands

Both the MarDx Marblot Western blot (Trevejo) dataset and the Immunetics QualiCode Western blot (510(k)) dataset reported IgG immunoblot results that included the 31- and 34-kDa bands in healthy controls; the latter dataset, however, reported only summary information on IgM immunoblot specificity utilizing standard CDC criteria. Table 4 displays random effect meta-analyses of IgG immunoblot FPRs associated with single-tier CDC criteria, Criteria A, and Criteria B. Modifying single-tier CDC IgG immunoblot criteria to include the 31- and 34-kDa bands led to modest but statistically significant loss of specificity (Table 4, footnote (b)).

FPRs for single-tier IgG immunoblots varied by both dataset and interpretive criteria. Tests positive by Criteria A, positive or equivocal by Criteria A, and positive or equivocal by Criteria B demonstrated statistically significant loss of specificity versus single-tier CDC criteria (Table 4, footnotes (c)–(e)); the 95% confidence intervals of these differences were wide for most comparisons due to data heterogeneity (I^2^ > 80%). In contrast to prior recommendations by Tilton et al. [28], we observed that reporting equivocal immunoblot results for Criteria B significantly eroded test specificity. The high FPRs associated with single-tier alternative interpretive criteria for IgG immunoblots in healthy controls stand in stark contrast to the 1% FPR reported for two-tiered CDC criteria for IgG immunoblots [5,26,40,49].

The MarDx Marblot Western blot (Trevejo [8]) dataset did not include samples from patients with potentially cross-reacting medical conditions; also, individual IgG immunoblot results were unavailable in the latter control group from the Immunetics QualiCode Western blot 510(k) dataset, preventing assessment of alternative immunoblot criteria in patients with potentially cross-reacting conditions. The FPRs of IgG immunoblots using alternative criteria might have been higher had sera from patients from the latter control group been included. Alternative criteria performance in patients with potentially cross-reacting conditions is, however, addressed in Section 3.6 using controls from the Viramed ViraStripe line blot (LSR) dataset.

### 3.3. Specificity of IgG and IgM Immunoblots That Exclude the 31- and 34-kDa Bands

By definition, equivocal IgG and IgM Western blots using Criteria A include the 31-kDa band (Table 1). The frequency of the 31- and 34-kDa IgG bands in healthy controls using the Immunetics QualiCode Western blot dataset was 15% and 11%, respectively. In contrast, the frequencies of the 31-and 34-kDa IgG band in studies of healthy controls by Ma et al. [50], Trevejo et al. [8], and Dressler et al. [34] were less than 3% each, raising concerns about band interpretation in the Immunetics QualiCode Western blot dataset. In order to assess the contributions of the 31- and 34-kDa bands to false-positive IgG immunoblots, we performed additional random effect meta-analyses of the Immunetics QualiCode Western blot and MarDx Marblot Western blot (Trevejo [8]) datasets without including the latter bands. One additional dataset, the Viramed ViraStripe line blot (LSR) dataset, was included because it contained sufficient individual IgG and IgM immunoblot data to permit analyses of alternative criteria, but did not utilize the 31- and 34-kDa bands. The meta-analyses in Table 5 demonstrate that the majority of false-positive IgG immunoblot results reported in Table 4 are unrelated to the 31- and 34-kDa bands; compared to single-tier CDC criteria, excess FPRs of 9.0% for IgG immunoblots positive by Criteria A (95% CI: 1.0% to 23.7%) and 14.4% for IgG immunoblots positive or equivocal by Criteria B (95% CI: 9.2% to 25.3%) were still observed in healthy controls. Also, single-tier IgG immunoblot specificity by CDC criteria using the Immunetics QualiCode Western blot dataset was consistent with other FDA-cleared immunoblot kits, arguing against over-reading of band intensity (see Supplementary Tables S1 and S2).

Meta-analyses of IgM immunoblot specificity utilized both the MarDx Marblot Western blot (Trevejo [8]) and Viramed ViraStripe line blot (LSR) datasets. We were unable to include the Immunetics QualiCode Western blot 510(k) dataset in this analysis because IgM immunoblot results were not available for individual control specimens, precluding assessment of alternative criteria. If the 31- and 34-kDa IgM bands are omitted, then single-tier IgM immunoblot performance is the same when using either Criteria A or CDC criteria (Table 5). IgM immunoblots positive or equivocal by single-tier Criteria B demonstrated a significantly higher FPR than single-tier CDC criteria among healthy controls; the composite difference in the FPR was 16.9% (95% CI: 12.4–21.8).

### 3.4. Individual and Combined IgG and IgM Immunoblot Performance: Comparison of CDC Criteria to Alternative Criteria That Include the 31- and 34-kDa Bands

We performed separate random effect meta-analyses of CDC IgG and IgM immunoblot criteria, applied to six different datasets, and compared composite sensitivity and specificity results to alternative Criteria A and B, applied only to dataset 4 (MarDx Marblot Western blots (Trevejo [8])); the latter dataset is the only one that reports 31- and 34-kDa band results for both IgG and IgM immunoblots. See Table 6 and Table 7 for IgG and IgM immunoblot performance, respectively. When utilizing CDC criteria, we observed a significant variation in both IgG and IgM immunoblot sensitivities among kits and among datasets using the same immunoblot kit. Individual IgG and IgM immunoblot sensitivities using CDC criteria for dataset 4 (MarDx Marblot Western blots (Trevejo [8])) appeared significantly lower than composite sensitivities observed utilizing the same MarDx Marblot IgG and IgM Western blot kits and CDC criteria for datasets 3 and 8 combined (derived using fixed effect meta-analyses). For IgG immunoblots, single-tier CDC criteria were positive in 19/120 (15.7%) early LD sera from dataset 4 versus 106/351 (30.2%) sera from datasets 3 and 8 combined (*p* = 0.0018 by two-tailed Fisher’s exact test). For IgM immunoblots, single-tier CDC criteria were positive in 46/120 (38%) early LD sera from dataset 4 versus 186/351 (53.0%) sera from datasets 3 and 8 combined (*p* = 0.006 by two-tailed Fisher’s exact test). IgG immunoblot specificities, interpreted using single-tier CDC criteria, were more homogeneous (i.e., consistent) by random effect meta-analysis (Table 6; I^2^ = 0) than IgM immunoblot specificities (Table 7; I^2^ = 86%).

Based on the meta-analyses in Table 6 and Table 7, no clear diagnostic advantage was observed for alternative Criteria A and B over the composite performance of either IgG or IgM immunoblots using single-tier CDC criteria. The latter comparison is limited because only the MarDx Marblot Western blot (Trevejo [8]) dataset was used to assess the performance of alternative criteria and 95% confidence intervals were wide for most results; however, IgG and IgM immunoblots positive or equivocal by single-tier Criteria B in this dataset demonstrated significantly higher FPRs than composite CDC criteria (i.e., their 95% confidence intervals did not overlap).

Sensitivity and specificity results related to the combined use of IgG and IgM immunoblotting in the MarDx Marblot Western blot (Trevejo [8]) dataset are reported in Table 8. Overall sensitivity for early LD was improved by only 0.8% for Criteria A and 1.7% for Criteria B by including the 31- and 34-kDa bands in the immunoblot panels. Although not detailed in Table 6, the sensitivity of CDC criteria in the above dataset would have improved by only 0.8% if they had been modified to include the 31- and 34-kDa bands (as previously reported by Trevejo et al. [8]). The sensitivity of single-tier CDC criteria reported in Table 8 was significantly less than immunoblots positive or equivocal by single-tier Criteria B (43.3% versus 72.5%, respectively, *p* < 0.0001 by two-tailed McNemar’s test), or positive by single-tier Criteria A (43.3% versus 54.2%, respectively, *p* = 0.0002 by two-tailed McNemar’s test). The single-tier FPR in healthy controls positive or equivocal by Criteria B was significantly higher than single-tier CDC criteria (31.6% versus 5.3%, respectively, *p* = 0.002 by two-tailed McNemar’s test), lowering its positive likelihood ratio and predictive value. The single-tier performance of Criteria B might have been even worse if potentially cross-reacting medical conditions had been included in the control population. All two-tiered criteria demonstrated 100% specificity for this dataset but suffered some loss of sensitivity for early LD (ranging from 7.5% for Criteria A to 17.5% for immunoblots positive or equivocal by Criteria B). Negative likelihood ratios were also slightly worse when utilizing two-tiered approaches. Nevertheless, significantly more LD samples were positive or equivocal by two-tiered Criteria B or positive by two-tiered Criteria A than standard two-tiered CDC criteria (Table 8, footnote (d)). The above data suggest potential value in using a second-tier EIA to confirm alternative immunoblot results; both single-tier and two-tiered approaches using alternative criteria are explored further using additional datasets in Section 3.5.

### 3.5. Combined Performance of IgG and IgM Immunoblots Using CDC Criteria and Alternative Criteria That Exclude the 31- and 34-kDa Bands

The meta-analyses presented in Table 9, Table 10 and Table 11 report the combined performance of IgG and IgM immunoblots using both single-tier and two-tiered approaches for CDC criteria, Criteria A, and Criteria B. Composite FPRs for single-tier IgG and IgM immunoblots using CDC criteria, Criteria A (only positive results), and Criteria B (either positive or equivocal results) were 6.1%, 12.4%, and 35%, respectively, in healthy controls, confirming the concerns about excessive FPRs associated with single-tier criteria. The composite FPRs for alternative criteria reported above did not include data from the Immunetics QualiCode Western blot (510(k)) dataset due to missing IgM immunoblot results; the high FPRs associated with IgG immunoblots alone from the latter dataset (Table 5, Table 9, Table 10 and Table 11) reinforce concerns about alternative criteria specificity. Even though the above IgG and IgM immunoblots did not utilize the 31- and 34-kDa bands, data from Table 4 and Table 5, as well as Supplementary Table S3 (Section B), argue that alternative criteria specificity would likely have been worse had the latter bands been included. Composite sensitivities for single-tier IgG and IgM immunoblots using CDC criteria, Criteria A (only positive results), and Criteria B (either positive or equivocal results) were 60.5%, 65.9%, and 72.5%, respectively.

Two-tiered approaches led to highly significant gains in specificity as well as concurrent losses in sensitivity compared to single-tier approaches for all three criteria. The composite improvements in specificity between single-tier and two-tiered approaches were 5.5% for CDC criteria (95% CI: 1.6% to 11.6%); 11.4% for assays positive by Criteria A (95% CI: 4.3% to 21.2%); and 30.9% for assays positive or equivocal by Criteria B (95% CI: 25.2% to 36.8%). The composite losses of sensitivity associated with two-tiered approaches were 10.9% for CDC criteria (95% CI: 7.4% to 15.0%); 7.5% for assays positive by Criteria A (95% CI: 4.3% to 11.5%); and 12.9% for assays positive or equivocal by Criteria B (95% CI: 5.0% to 23.7%).

Point estimates of likelihood ratios based on composite sensitivity and FPRs demonstrated a 4.6- to 10.2-fold improvement in positive likelihood ratios for alternative criteria using a two-tiered approach. Despite the sensitivity loss associated with two-tiered approaches for alternative criteria, negative likelihood ratios were reduced by only 3% for Criteria A and 1% for Criteria B. These observations suggest that applying a two-tiered approach to alternative criteria significantly improved their ability to confirm disease but only marginally reduced their ability to exclude LD; the above suggestion should be tempered by the wide confidence intervals associated with composite changes in both sensitivities and specificities for two-tiered alternative criteria. Also, patients with potentially cross-reacting medical conditions were not included in the specificity calculations reported above.

Two-tiered CDC criteria employing immunoblots (Table 9) appeared less sensitive but more specific than two-tiered alternative Criteria A and B (Table 10 and Table 11), predicting that two-tiered CDC criteria may be better at confirming disease when pretest risk is low and two-tiered alternative interpretive criteria may be better at excluding disease when pretest risk is high; the latter concept is explored further in Section 3.6.

### 3.6. Comparative Performance of Two-Tiered CDC Criteria Using Immunoblots, Modified Two-Tiered Criteria, and Alternative Immunoblot Criteria Using the CDC Lyme Serum Repository

Utilizing the Viramed ViraStripe line blot (LSR) dataset, we report the performance of two-tiered CDC criteria using both IgG and IgM immunoblots, two different MTT (all-EIA) approaches, and alternative immunoblot criteria (Table 12). Because the latter dataset includes controls from individuals with potentially cross-reacting medical conditions, we believe these results may better reflect real-world performance of the above interpretive criteria. Applying Equation (2) from the Methods section, we also report the minimum pretest probability of LD required for a test positive by a given criterion to be correct at least 50% of the time (i.e., demonstrates a positive predictive value ≥50%); the latter represents a decision threshold for the clinical application of that criterion.

As previously noted, most serologic tests for LD in the US are performed in a setting of low pretest risk (<5%) [12,13,14]. European studies have reported similar results among individuals with non-specific symptoms [51]. Although CLIA-certified laboratories that offer laboratory-developed immunoblots predominantly utilize single-tier interpretive Criteria A and B [32,33], only tests positive or equivocal by two-tiered criteria listed in Table 12 were likely to generate PPVs > 50% when the pretest risk of LD was <10%.

Similar to the results reported above using the MarDx Marblot Western blot (Trevejo [8]) dataset (Table 8), the sensitivities of both single-tier and two-tiered alternative Criteria A and B using the Viramed ViraStripe line blot (LSR) dataset were greater than that of two-tiered CDC immunoblot criteria (Table 12). The specificity of two-tiered CDC immunoblot criteria was 2.0% greater than immunoblots positive by two-tiered Criteria A and 5.1% greater than immunoblots positive or equivocal by two-tiered Criteria B (*p* = 0.0156 and *p* < 0.0001, respectively, by two-tailed McNemar’s test); the differences in specificity between two-tiered CDC immunoblot criteria and the single-tier alternative criteria listed in Table 12 were even larger. As mentioned in Supplementary File S2, the duration of illness for individual LD patients and controls from the LSR was not available in the dataset provided by the CDC; alternative criteria therefore utilized both IgG and IgM immunoblot results for all controls. Utilizing a presumptive duration of illness > 30 days for two control samples from patients with rheumatoid arthritis with positive or equivocal IgM immunoblot results by Criteria B (Table 12, footnote (f)), we evaluated the impact of recategorizing these samples as negative by Criteria B; recategorization would not have significantly altered assay specificity or statistical comparisons with CDC criteria.

Faced with choosing between either a more sensitive or more specific assay, we utilized Equation (1) from the Methods section to calculate the pretest risk of LD wherein the accuracy of alternative immunoblot criteria would exceed that of two-tiered CDC immunoblot criteria. The pretest risk of Lyme disease would need to exceed 37.1% for tests positive by single-tier Criteria A, 55.9% for tests positive or equivocal by single-tier Criteria B, 12.5% for tests positive by two-tiered Criteria A, and 22.4% for tests positive or equivocal by two-tiered Criteria B for the accuracy of the above criteria to exceed that of two-tiered CDC immunoblot criteria. Based on the above analysis, two-tiered CDC immunoblot criteria appear preferable in most clinical settings.

The specificity of the MTT using the Zeus VlsE1/pepC10 EIA Test System was 4.4% greater than immunoblots positive by two-tiered Criteria A and 7.1% greater than immunoblots positive or equivocal by two-tiered Criteria B (*p* = 0.01 and *p* = 0.0002, respectively, by two-tailed Fisher’s exact test); the differences in specificity between the MTT using the Zeus VlsE1/pepC10 EIA Test System and the single-tier alternative immunoblot criteria listed in Table 12 were even larger. The MTT using the Zeus VlsE1/pepC10 Test System demonstrated the same sensitivity for early LD as immunoblots positive or equivocal by single-tier Criteria B (83.3%), the most sensitive of all alternative criteria; both positive and negative likelihood ratios for the Zeus MTT were superior to all other criteria listed in Table 12. As previously noted by Biggerstaff [47], diagnostic tests that demonstrate superior positive and negative likelihood ratios than a competing assay are superior regardless of the pretest risk of disease. The confidence intervals for the above likelihood ratios were wide, tempering the above comparisons.

Because the alternative immunoblot criteria evaluated using the Viramed ViraStripe line blot (LSR) dataset did not utilize the 31- and 34-kDa bands, we potentially underestimated their sensitivity for early LD; although we believe that the latter risk is low, we performed one-way sensitivity analyses to estimate the performance of the most sensitive alternative criteria had the 31- and 34-kDa bands been included. We first assumed positive likelihood ratios for alternative criteria at the upper-bound of their 95% confidence intervals for the Viramed ViraStripe line blots (LSR, dataset 8) (i.e., 5.4 for tests positive by single-tier Criteria A and 2.7 for tests positive or equivocal by single-tier Criteria B). For the purpose of our sensitivity analysis, we also assumed that immunoblot specificity would remain unchanged after including the 31- and 34-kDa bands; the 95% upper-bound of the positive likelihood ratio for each alternative criterion is therefore mathematically equivalent to assuming immunoblot sensitivity that exceeds 97% for both Criteria A and B. Utilizing Equation (2) from the Methods section, we then calculated the pretest risk of LD needed to generate a PPV of 50% for each alternative immunoblot criterion: 15.6% for tests positive by single-tier Criteria A and 27.0% for tests positive or equivocal by single-tier Criteria B. The latter thresholds are only slightly improved compared to the baseline estimates in Table 12 for these two alternative criteria. Based on the above sensitivity analyses, substantial pretest risk would still have been required to ensure test accuracy using alternative criteria, even after including the 31- and 34-kDa bands.

The LSR includes 46 sera from patients with disseminated and late-stage LD, including 7 patients with Lyme carditis, 10 patients with Lyme neuroborreliosis, and 29 patients with Lyme arthritis. Among these 46 later-stage sera, the lowest sensitivity was noted in immunoblots positive by Criteria B (87.0% using a two-tiered approach and 89.1% using a single-tier approach). All other criteria, including CDC-advocated criteria, demonstrated sensitivity greater than 95% in sera from patients with later-stage disease, whether using a single-tier or two-tiered approach. Sensitivity was 100% in later-stage samples that were positive by single-tier Criteria A, positive or equivocal by single-tier Criteria B, or positive by the MTT using the Zeus VlsE1/pepC10 EIA Test System. Since the studies that used LSR specimens do not include the 31- and 34-kDa bands, the already high sensitivity for disseminated and late-stage LD demonstrated by both standard and alternative criteria argues that adding these bands to the immunoblot panel may not be diagnostically necessary.

## 4. Discussion

Alternative immunoblot criteria have been proposed because of concern about the sensitivity of antibody assays for LD diagnosis, particularly for early-stage disease [29,30]. There is a paucity of peer-reviewed literature concerning alternative immunoblot criteria [29,30,40]. We present previously unpublished data from three FDA-cleared immunoblot test kits, comparing CDC immunoblot criteria to two alternative immunoblot criteria in 198 sera from patients with early LD, 46 sera from patients with disseminated and late-stage infection, 144 sera from patients with potentially cross-reacting medical conditions, and 670 healthy controls. We also compared CDC-advocated MTT criteria to alternative immunoblot criteria using data from the CDC Lyme Serum Repository and performed a meta-analysis of the performance of CDC immunoblot criteria using additional commercially available immunoblot test kits. Our analyses raise significant concerns about the specificity of alternative immunoblot criteria.

We were unable to prove diagnostic benefit from including the 31- and 34-kD bands in the immunoblot panel; utilizing the MarDx Marblot Western blot (Trevejo) dataset, we observed that including these two bands in both IgG and IgM immunoblot panels increased sensitivity for early LD by only 0.8% for Criteria A and 1.7% for Criteria B. Even without including the 31- and 34-kDa bands, immunoblots positive by Criteria A and immunoblots positive or equivocal by Criteria B demonstrated >95% sensitivity in sera from patients with disseminated and late-stage LD from the CDC Lyme Serum Repository (dataset 8). Our findings are consistent with prior studies that observed that antibody responses to OspA and OspB antigens develop principally in later stages of LD, a time when the immune response is already broad and mature [34,35].

Instead, we observed more frequent false-positive IgG immunoblots (Table 4) after including the 31- and 34-kDa bands; we noted significantly higher composite FPRs in healthy controls after adding these bands to CDC interpretive criteria (5.7% versus 2.1%, respectively, when utilized as single-tier assays). Meta-analyses of IgG immunoblot performance reported in Table 4 demonstrated composite FPRs of 17.2% for immunoblots positive or equivocal by single-tier Criteria A and 23.9% for immunoblots positive or equivocal by single-tier Criteria B. As demonstrated in Table 5, the majority of these false-positive IgG immunoblots were due to IgG band combinations that did not include the 31- and 34-kDa bands. A high composite FPR (16.9%) was also noted for IgM immunoblots positive or equivocal by single-tier Criteria B without including the latter two bands (Table 5). The above observations argue that the high FPRs associated with immunoblots interpreted using alternative criteria are largely a consequence of accepting fewer bands to consider a test positive, reporting equivocal results, and employing a single-tier approach. Prior studies of alternative immunoblot performance utilized controls that were “negative for antibodies to *B. burgdorferi*” [31] and “known to be negative for Lyme disease” [30] before evaluating alternative immunoblot techniques; prescreened controls would likely demonstrate a lower FPR using alternative criteria than unscreened controls, potentially explaining at least some of the differences between our results and prior investigations.

Meta-analyses of single-tier immunoblots positive by alternative Criteria A and positive or equivocal by Criteria B (Table 10 and Table 11) demonstrated high composite FPRs using combined IgG and IgM immunoblotting, even without utilizing the 31- and 34-kDa bands. Although both alternative immunoblot criteria demonstrated significantly better specificity when using a two-tiered approach, they were still less specific than two-tiered CDC criteria in healthy controls (Table 9). Additional analyses that included controls with potentially cross-reacting medical conditions (Table 12) indicated that the pretest risk of LD required for alternative criteria to demonstrate accuracy equivalent to two-tiered CDC immunoblot criteria was 12.5% for tests positive by two-tiered Criteria A and 22.4% for tests positive or equivocal by two-tiered Criteria B (Results, Section 3.6).

There are only limited circumstances where the pretest risk of LD exceeds 10%. Erythema migrans (EM) is a clinical diagnosis that does not require serology except when atypical in appearance (e.g., ulcerated or vesicular skin lesions); a large prospective study demonstrated that these atypical EM presentations account for less than 10% of U.S. cases [52]. A 2011 prospective study by Garro et al. [53] found that 13.3% of US children from endemic communities with aseptic meningitis had LD, although the incidence was 27% when concurrent facial palsy was present. A prospective study by Ljostad et al. [54] determined that only 10% of European adults who developed facial palsy in endemic communities had LD. Newly diagnosed oligo-articular arthritis in highly endemic communities was due to LD in 6% to 12% of adults and 31% to 47% of children [55,56,57,58]. Even in tertiary LD referral clinics located in endemic communities, overall pretest risk ranged from 9.6% to 14.6%; most diagnosed with LD demonstrated objective physical findings [16,59,60]. Thus, in most practice settings, two-tiered CDC immunoblot criteria will be more accurate than alternative criteria.

Even in clinical settings where the pretest risk of LD exceeds 10%, our data suggest that CDC-advocated MTT approaches may be preferable to alternative immunoblot criteria because of equivalent sensitivity and superior specificity; the PPV of the Zeus MTT assay was greater than 50% when pretest risk of LD was greater than 1.8%. Two new MTT assays have recently been FDA-cleared: the Liaison Lyme Total Antibody Plus kit with confirmation by either the Liaison Lyme IgG or Liaison Lyme IgM test kits (Diasorin Inc., 510(k) applications K202574, K202573, and K193051) and the Viramed Borrelia All-In-One ViraChip Test Kit (Viramed Biotech AG, 510(k) application K220016). These two additional MTTs demonstrate performance characteristics similar to the MTT that uses the Zeus VlsE1/pepC10 EIA Test System (80% to 90% sensitive and 96% to 98% specific), providing additional alternatives to immunoblot assays.

Recombinant line blots, as proposed by Liu et al. [30], hold promise for improved performance compared to traditional Western blots [24], but methodological issues, such as antigen choice and concentration, interpretive criteria, and standardization practices, provide challenges to their adoption as an alternative to CDC-advocated approaches. Also, utilizing recombinant antigens for line blots does not guarantee specificity. Epitopes from multiple recombinant antigens from *B. burgdorferi* sensu stricto, including OppA-2 (p58), FlaB (p41), OspC (p23), DbpA (p18), and β-3 integrin binding protein (p66), can bind antibodies that cross-react with other medical conditions [61,62,63,64,65]. Western blots and recombinant-based line blots for diagnosis of US and European LD demonstrate cross-reacting antibodies to leptospirosis, *Helicobacter pylori*, syphilis, Epstein–Barr virus, BK virus, and cytomegalovirus [66,67,68]. Antigens purified by electrophoresis and chromatography have also been utilized in line blots, but again demonstrate the potential for cross-reacting antibodies [5].

There are several limitations to the current paper. We evaluated alternative immunoblot criteria used to interpret laboratory-developed tests, but not the laboratory-developed tests themselves. We instead assessed the performance of alternative interpretive criteria using two FDA-cleared Western blots and one FDA-cleared line blot; it is possible that the performance of these FDA-cleared immunoblots may differ from the laboratory-developed immunoblots. Differences in subjective interpretation of weak-positive bands may also contribute to differences in assay performance [10]. Nevertheless, one prior study in 2014 by Fallon et al. [40] suggested that laboratory-developed IgG Western blots and standard kits demonstrated similar performance when using the same CDC interpretive criteria. The Immunetics QualiCode Western blot, MarDx Marblot Western blot, and Viramed ViraStripe line blot test kits are no longer commercially available for comparison to laboratory-developed immunoblots for IgG and IgM antibodies to *B. burgdorferi*. Because of high FPRs observed when applying alternative interpretive criteria to the above datasets, it is important that laboratories employing alternative immunoblot criteria demonstrate both rigorous standardization protocols and clinical studies using well-characterized samples before claiming equivalence to CDC immunoblot criteria.

The Sfeir study [3] of the Zeus MTT assay reported results for most but not all samples from the CDC Lyme Serum Repository (LSR). The CDC provides blinded samples from the LSR to device manufacturers in stages during the assay development process [69]; the results reported in Table 12 from Sfeir et al. [3] represent the remaining, premarketing validation set of early LD samples and controls. It is therefore likely that LSR validation sample set reported above is representative of the overall LSR collection.

Because assay sensitivity and FPRs are typically positively correlated, univariate meta-analyses of sensitivity and FPRs may slightly under-estimate their performance relative to bivariate analyses; the difference between these two methods is usually minor for each parameter (less than 2%) and is unlikely to affect comparisons with alternative criteria, particularly when the differences between criteria are greater than 4% for specificity or 6% for sensitivity [43]. All major comparisons of specificity between CDC and alternative criteria identified differences that exceeded the above limits, arguing in favor of true differences in performance.

Immunoblot performance using either CDC or alternative interpretive criteria demonstrated significant heterogeneity, favoring use of random effect meta-analyses; the latter analytic choice led to wide confidence intervals for many composite parameters. We nevertheless identified statistically significant differences in both IgG and IgM immunoblot specificity between CDC criteria and alternative interpretive criteria in healthy controls. Data from the CDC Lyme Serum Repository also identified high FPRs using alternative criteria in individuals with potentially cross-reacting medical conditions.

The pretest risk of LD not only helps determine when to order diagnostic tests, but also helps choose between assays in different clinical settings based on their respective sensitivities and specificities. We did not perform a formal decision-analysis to identify the pretest risk of LD where alternative immunoblot criteria might be preferred to CDC-advocated criteria. Alternative immunoblot criteria were generally more sensitive but less specific than two-tiered CDC immunoblot criteria. Choosing between different assays necessitates tradeoffs between false-positive and false-negative test results; the magnitude of these tradeoffs depends on the pretest risk of LD and the relative harm associated with each choice. While there is obvious harm associated with false-negative LD serology, the injuries associated with false-positive LD serology may be less visible but equally serious. Numerous studies have documented that patients with other treatable diseases have been misdiagnosed with LD because of erroneous serology [59,60,70,71]. Extensive overtreatment with antibiotics for LD has also been described [60,70], sometimes with life-threatening consequences [72,73]. Due to the difficulty calculating the harm from false-positive serology, we utilized overall test accuracy to choose between assays in different clinical settings and identified the pretest risk of LD that yielded a PPV ≥50% for a given criterion as a reasonable decision threshold for that criterion.

Although we focused our analyses on alternative interpretive criteria used for laboratory-developed immunoblots, other possible modifications of immunoblot criteria warrant mention. Western blot assays utilize only in vitro expressed antigens. Neither the laboratory-developed immunoblots discussed in the current manuscript nor our current study clinically assessed the value of including *B. burgdorferi* antigens that are predominantly expressed in vivo, such as VlsE. Some European recombinant line blots include the latter antigen [5,24], but none have been FDA-cleared in the US. The current study also did not evaluate disease stage-specific immunoblot criteria, as suggested by Hauser et al. [74] and Robertson et al. [75]. The breadth of the immune response provided by immunoblotting may provide a window to help answer the latter question, but, as recognized by Liu et al. [30], large prospective studies would be required to optimize disease-stage specific diagnosis using immunoblot techniques. Although immunoblot responses can be quantified using densitometry and multivariate algorithms [41,76], multiplex technologies for antibody detection using microsphere and plasmonic biochip techniques offer a broader dynamic range than immunoblotting and greater reproducibility [77,78,79]. The availability of highly sensitive and reproducible multiplex techniques argues that prospective studies using these newer technologies might be more fruitful than employing an older methodology.

## 5. Conclusions

Our data confirm the results reported by Fallon et al. [40] concerning worrisome FPRs for alternative IgG and IgM immunoblot interpretive criteria. The more liberal nature of alternative criteria and their single-tier approach, rather than the inclusion of the 31- and 34-kDa bands, appear most responsible for the loss of specificity relative to CDC criteria. The sensitivity of alternative immunoblot interpretive criteria for early LD was not significantly enhanced by including the 31- and 34-kDa bands and it is doubtful that these antigens play a significant role in routine diagnosis of later-stage disease. The sensitivity of modified two-tiered (MTT), all-EIA assays for early LD was comparable to alternative immunoblot criteria and superior to that of standard two-tiered serology using immunoblots. MTT assays also demonstrated superior specificity compared to single-tier alternative immunoblot criteria. The recent availability of MTT assays may therefore limit the impetus to employ alternative immunoblot criteria to enhance disease detection. Due to limited standardization and high false-positive rates, the presently evaluated single-tier alternative immunoblot interpretive criteria appear inferior to CDC two-tiered criteria.

## Figures and Tables

**Table 1:** Alternative Criteria A for interpretation of Western blots and line blots ^a,b^.

Method	Positive	Equivocal	Negative or Exception
IgG Western blot bands (kDa) ^c^	At least 2 of 6 bands present: 23/25, 31, 34, 39, 41, and 83/93	Only 31 and 41 bands present	Either 1 or no bands present
IgM Western blot bands (kDa) ^c^	At least 2 of 6 bands present: 23/25, 31, 34, 39, 41, and 83/93 (see exception)	Only 31 and 83/93 bands present OR Only 31 and 41 bands present	Either no bands OR only 41 and 83/93 bands present
IgG line blot bands (kDa)	At least 2 of 6 bands present: 23, 31, 34, 39, 41, and 93	NA	Either 1 or no bands present
IgM line blot bands (kDa)	At least 2 of 5 bands present: 23, 31, 34, 39, and 41	NA	Either 1 or no bands present

NA, not applicable; kDa, kilodalton. ^a^ Adapted from studies by Shah et al. [29,31] and Liu et al. [30] as a single-tier diagnostic approach by CLIA-certified Laboratory A [32] for all stages of Lyme disease. Shah [29] utilized whole-cell lysate from both the B31 and 297 strains of *B. burgdorferi* for Western blotting. Line blots advocated by Liu [30] utilized recombinant antigens from 4 European and 4 North American strains or genospecies of *B. burgdorferi* sensu lato. ^b^ Laboratory A recommends resolving equivocal results either by testing using an alternative immunoassay, such as a laboratory-developed immunoblot utilizing recombinant OspA, or repeating the immunoblot in 6 to 8 weeks. ^c^ Laboratory A recognizes IgG and IgM Western blot bands observed between 23- and 25-kDa and between 83- and 93-kDa as diagnostically significant. See text regarding the variable migration of p23 and p93 on Western blot.

**Table 2:** Alternative Criteria B for interpretation of immunoblots ^a,b^.

Method	Positive	Equivocal	Negative
IgG immunoblot bands (kDa)	At least 3 of 5 bands present: 23, 31, 34, 39, and 93	Either 1 or 2 bands present	No bands present
IgM immunoblot bands (kDa)	At least 2 of 5 bands present: 23, 31, 34, 39, and 41	1 band present	No bands present

^a^ Adapted from criteria developed by Tilton et al. [28] as a single-tier diagnostic approach by CLIA-certified Laboratory B [33]. To identify differences between criteria used by Laboratory B and Tilton et al. [28] see document dated 14 November 2019 from Laboratory B in Supplementary File S1; the latter criteria have been used for both Western blots and line blots. ^b^ Although Laboratory B [33] recommends confirmation of positive or equivocal EIA results by immunoblotting, they also employ immunoblotting as a single-tier diagnostic approach, reporting immunoblot results using both CDC criteria and alternative Criteria B. Only IgG immunoblots are recommended by Laboratory B for diagnosis of late Lyme disease (Supplementary File S1). If LD is clinically suspected despite negative or equivocal immunoblot results, then repeating the immunoblot within 2 to 4 weeks is recommended by Laboratory B. Methods to resolve equivocal immunoblot results were not addressed by Tilton et al. [28].

**Table 3:** Datasets used for analysis of immunoblots employing the B31 strain of *B. burgdorferi* ^a,b^.

Dataset No.	Dataset Description
1	Immunetics QualiCode *B. burgdorferi* IgG and IgM Western blot kits (510(k))
2	EUROIMMUN Anti-*Borrelia burgdorferi* US IgG and IgM Western blot kits (510(k))
3	MarDx *B. burgdorferi* Marblot Strip Test System, IgG and IgM Western blot kits (510(k))
4	MarDx *B. burgdorferi* Marblot Strip Test System, IgG and IgM Western blot kits (Trevejo et al. [8])
5	MarDx *B. burgdorferi* Marblot Strip Test System, IgG and IgM Western blot kits (Johnson et al. [26])
6	Trinity Biotech Lyme *B. burgdorferi* MarStripe Tests (IgG and IgM line blot kits) (510(k))
7	Gold Standard Diagnostics *Borrelia burgdorferi* B 31 IgG and IgM Line Blot Test Kits (510(k))
8	Viramed Biotech AG *Borrelia* B31 ViraStripe IgG and IgM line blots and MarDx *B. burgdorferi* Marblot Strip Test System, IgG and IgM Western blot kits using the CDC Lyme Serum Repository (Molins et al. [5])

^a^ 510(k) is a premarket notification made to the FDA by a medical device manufacturer to demonstrate that a new device is as safe and effective as an existing, legally marketed device (i.e., substantially equivalent). Searchable FDA database: https://www.accessdata.fda.gov/scripts/cdrh/cfdocs/cfPMN/pmn.cfm (accessed on 1 June 2023) contains summary data submitted in support of premarket notification. ^b^ See Supplementary File S2 for full dataset descriptions.

**Table 4:** Comparative false-positive rates of single-tier immunoblots for IgG antibodies to *B. burgdorferi* using CDC criteria versus alternative criteria that include the 31- and 34-kDa bands ^(a)^.

Dataset	CDC without 31- and 34-kDa Bands Pos (%)	CDC with 31- and 34-kDa Bands Pos (%) ^b^	Criteria A Pos (%) ^c^	Criteria A Pos/Eq (%) ^d^	Criteria B Pos (%)	Criteria B Pos/Eq (%) ^e^
Immunetics QualiCode IgG WB						
Cross-reacting diseases	12/172 (7.0)	NA	NA	NA	NA	NA
Healthy endemic controls	2/278 (0.7)	14/278 (5.0)	82/278 (29.5)	99/278 (35.6)	9/278 (3.2)	101/278 (36.3)
Healthy non-endemic controls	6/151 (4.0)	11/151 (7.3)	46/151 (30.5)	57/151 (37.8)	9/151 (6.0)	59/151 (39.1)
Total for healthy controls	8/429 (1.9)	25/429 (5.8)	128/429 (29.8)	156/429 (36.4)	18/429 (4.2)	160/429 (37.3)
Total for all controls	20/601 (3.3)	NA	NA	NA	NA	NA
MarDx Marblot IgG WB (Trevejo)						
Potentially cross-reacting diseases	NA	NA	NA	NA	NA	NA
Healthy endemic controls	1/38 (2.6)	1/38 (2.6)	1/38 (2.6)	1/38 (2.6)	0/38 (0)	4/38 (10.5)
Random effect meta- analysis of FPR in healthy controls (95% CI)	2.1 (1.0–3.6)	5.7 (3.8–8.0)	14.7 (0.03–8.4)	17.2 (0.03–58.5)	2.8 (0.2–7.9)	23.9. (4.2–53.0)

kDa, kilodalton; Pos, proportion positive; Pos/Eq, proportion either positive or equivocal; FPR, false-positive rate; WB, Western blot; NA, not available; CI, confidence interval. ^a^ Criteria A and B include the 31- and 34-kDa IgG bands. ^b^ CDC criteria were modified to include the 31- and 34-kDa bands, such that any 5 of 12 bands were considered positive. The composite difference in FPR between modified and standard CDC criteria was 2.7% in healthy controls (95% CI: 0.3–7.4). See the Methods section for analytic details. ^c^ The composite difference in FPRs between assays positive by Criteria A and standard CDC criteria was 10.2% in healthy controls (95% CI: 2.0–50.8). ^d^ The composite difference in FPRs between assays positive or equivocal by Criteria A and standard CDC criteria was 12.4% in healthy controls (95% CI: 8.1–61.0). ^e^ The composite difference in FPRs between assays positive or equivocal by Criteria B and standard CDC criteria was 21.3% in healthy control (95% CI: 2.3 to 52.1).

**Table 5:** Meta-analyses of single-tier IgG and IgM immunoblot false-positive rates by CDC criteria, Criteria A, and Criteria B that exclude the 31- and 34-kDa bands.

Dataset	Criteria A Pos (%) ^a^	Criteria B Pos/Eq (%)	CDC Criteria Pos (%)
IgG Immunoblots			
Immunetics QualiCode WB	107/429 (24.9)	109/429 (25.4)	8/429 (1.9)
MarDx Marblot WB (Trevejo)	1/38 (2.6)	4/38 (10.5)	1/38 (2.6)
Viramed ViraStripe LB (LSR)	23/203 (11.3)	35/203 (17.2)	6/203 (3.0)
Random effect meta-analysis of IgG FPR (95% CI)	12.9% (3.9–26.1)	19.3% (12.4–27.3)	2.4% (1.4–3.7)
Meta-analysis of difference in IgG FPR versus CDC criteria ^b^ (95% CI)	9.0% (1.0–23.7)	14.4% (9.2–25.3)	NA
IgM Immunoblots			
MarDx Marblot WB (Trevejo)	1/38 (2.6)	8/38 (21.1)	1/38 (2.6)
Viramed ViraStripe LB (LSR)	16/203 (7.9)	49/203 (24.1)	16/203 (7.9)
Random effect meta-analysis of IgM FPR (95% CI)	7.1% (3.8–11.3)	23.9% (18.7–29.4)	7.1% (3.8–11.3)
Meta-analysis of difference in IgM FPR versus CDC criteria (95% CI) ^b^	0.2% (0–1.1)	16.9% (12.4–21.8)	NA

Pos, proportion positive; Pos/Eq, proportion either positive or equivocal; FPR, false-positive rate; CI, confidence interval; WB, Western blot; LB, line blot; LSR, CDC Lyme Serum Repository; NA, not applicable. ^a^ No equivocal specimens were observed using Criteria A when the 31- and 34-kDa bands were omitted. ^b^ A random effect meta-analysis utilized the differences in FPRs between a given alternative criterion and CDC criteria for each dataset, expressed as a proportion of the control population for that dataset, to generate a composite difference in FPR. See the Methods section.

**Table 6:** Comparative performance of single-tier CDC IgG immunoblot criteria to alternative criteria that include the 31- and 34-kDa bands ^a^.

Criteria	Study (Dataset No.) ^b^	Sensitivity (%) ^c^	FPR (%) ^d^	LR (+)	LR (−)
CDC	Immunetics QualiCode WB (1)	50/107 (46.7)	8/429 (1.9)	25.1	0.54
	EUROIMMUN WB (510(k)) (2)	19/34 (55.9)	3/198 (1.5)	37.0	0.45
	MarDx Marblot WB (510(k)) (3)	84/273 (30.8)	6/514 (1.2)	26.3	0.70
	MarDx Marblot WB (Trevejo) (4)	19/120 (15.8)	1/38 (2.6)	6.0	0.86
	MarDx Marblot WB (LSR) (8)	22/78 (28.2)	2/203 (1.0)	28.6	0.73
	Trinity MarStripe LB (510(k)) (6)	5/32 (15.6)	1/219 (0.5)	34.2	0.85
	Gold Standard LB (510(k)) (7)	3/40 (7.5)	2/234 (0.9)	8.8	0.93
	Viramed ViraStripe LB (LSR) (8)	19/78 (24.4)	6/203 (3.0)	8.2	0.78
Meta-analyses of CDC criteria ^e^ (95% CI)	Above studies except MarDx Marblot WB (LSR) (8) and Trinity MarStripe LB (510(k)) (6)	28.9% (17.9–41.3)	1.7% (1.1–2.4)	17.0	0.72
Criteria A (95% CI)	MarDx Marblot WB (Trevejo) (4) (Pos)	40/120 (33.3) (25.5–42.2)	1/38 (2.6) (0–13.5)	12.7 (1.8–89.1)	0.69 (0.60–0.79)
Criteria B (95% CI)	MarDx Marblot WB (Trevejo) (4) (Pos)	5/120 (4.2) (1.8–9.4)	0/38 (0) (0–9.1)	∞	0.96 (0.92–1.00)
	MarDx Marblot WB (Trevejo) (4) (Pos/Eq)	44/120 (36.7) (28.6–45.6)	4/38 (10.5) (4.2–24.1)	3.5 (1.3–9.1)	0.71 (0.60–0.84)

Legend: no., number; FPR, false-positive rate; LR (+), positive likelihood ratio; LR (−), negative likelihood ratio; ∞, infinity; WB, Western blot; LB, line blot; 510(k), medical device application to FDA (see description in Table 3); LSR, CDC Lyme Serum Repository; Pos, proportion positive; Pos/Eq, proportion either positive or equivocal. ^a^ Criteria A and B both utilize the 31- and 34-kDa bands, but no equivocal results were observed using Criteria A. ^b^ See Table 3 and Supplementary File S2 for dataset descriptions. ^c^ Sensitivity for early Lyme disease. ^d^ FPR based on results in healthy controls from both endemic and non-endemic areas. ^e^ The MarDx Marblot WB (LSR) used the same dataset as the Viramed ViraStripe LB (LSR). The MarDx Marblot WB (510(k)) controls may have overlapped with controls used for the Trinity MarStripe LB (510(k)) (i.e., both manufactured by Trinity Biotech USA, Jamestown, NY, USA). Both the MarDx Marblot WB (LSR) and Trinity MarStripe LB (510(k)) were therefore excluded from the meta-analyses due to data overlap. Point estimates of likelihood ratios from composite sensitivity and specificity results for CDC criteria were reported for heuristic comparisons to other criteria.

**Table 7:** Comparative performance of single-tier CDC IgM immunoblot criteria to alternative criteria that include the 31- and 34-kDa bands ^a^.

Criteria	Study (Dataset No.) ^b^	Sensitivity (%) ^c^	FPR (%) ^d^	LR (+)	LR (−)
CDC	Immunetics QualiCode WB (1)	88/99 (88.9)	19/430 (4.4)	20.1	0.12
	EUROIMMUN WB (510(k)) (2)	19/34 (55.9)	9/198 (4.5)	12.3	0.47
	MarDx Marblot WB (510(k)) (3)	152/273 (55.7)	27/514 (5.3)	10.6	0.48
	MarDx Marblot WB (Trevejo) (4)	46/120 (38.3)	1/38 (2.6)	14.6	0.63
	MarDx Marblot WB (LSR) (8)	34/78 (43.6)	4/203 (2.0)	22.1	0.58
	Trinity MarStripe LB (510(k)) (6)	14/29 (48.3)	1/220 (0.5)	106.2	0.52
	Gold Standard LB (510(k)) (7)	35/40 (87.5)	0/234 (0)	∞	0.13
	Viramed ViraStripe LB (LSR) (8)	47/78 (60.3)	16/203 (7.9)	7.5	0.45
Meta-analyses of CDC criteria ^e^ (95% CI)	Above studies except MarDx Marblot WB (LSR) (8) and Trinity MarStripe LB (510(k)) (6)	65.3% (48.4–80.4)	3.9% (1.6–7.0)	16.7	0.36
Criteria A (95% CI)	MarDx Marblot WB (Trevejo) (4) (Pos)	48/120 (40.0) (31.7–48.9)	1/38 (2.6) (0–13.5)	15.2 (2.2–106.5)	0.62 (0.53–0.72)
Criteria B (95% CI)	MarDx Marblot WB (Trevejo) (4) (Pos)	47/120 (39.2) (30.9–48.1)	1/38 (2.6) (0–13.5)	14.9 (2.1–104.3)	0.63 (0.54–0.73)
	MarDx Marblot WB (Trevejo) (4) (Pos/Eq)	80/120 (66.7) (57.8–74.5)	9/38 (23.7) (13.0–39.2)	2.8 (1.6–5.1)	0.44 (0.32–0.60)

See Table 6 legend for abbreviations. ^a^ Both Criteria A and B utilize the 31- and 34-kDa bands, but no equivocal results were observed using Criteria A. ^b^ See Table 3 and Supplementary File S2 for dataset descriptions. ^(c)^ Sensitivity for early Lyme disease. ^d^ FPR based on results in healthy controls from both endemic and non-endemic areas. ^e^ Meta-analyses of CDC IgM immunoblot kit performance excluded MarDx Marblot WB (LSR) and Trinity MarStripe LB (510(k)) datasets due to overlap with other datasets. See explanation in Table 6. Point estimates of likelihood ratios using composite sensitivity and FPR for CDC criteria were reported for heuristic comparisons to other criteria.

**Table 8:** Combined IgG and IgM immunoblot performance in MarDx Marblot Western blot (Trevejo [8]) dataset for diagnosis of early Lyme disease: single-tier and two-tiered approaches using CDC and alternative interpretive criteria ^a^.

Immunoblot Criteria	Test Result ^b^	Sensitivity IgG (%)	Sensitivity IgM (%)	Combined Sensitivity (%)	Combined FPR (%) ^c^	LR (+) (95% CI)	LR (−) (95% CI)
Criteria A	Pos	41/120 (34.2)	48/120 (40.0)	65/120 (54.2)	2/38 (5.3)	10.3 (2.6–40.1)	0.48 (0.39–0.60)
	Pos (two-tiered)	36/120 (30.0)	44/120 (36.7)	56/120 (46.7)	0/38 (0)	∞	0.53 (0.45–0.63)
Criteria B	Pos	5/120 (4.2)	47/120 (39.2)	50/120 (41.7)	1/38(2.6)	15.8 (2.3–110.8)	0.60 (0.51–0.70)
	Pos/Eq	44/120 (36.7)	80/120 (66.7)	87/120 (72.5)	12/38 (31.6)	2.3 (1.4–3.7)	0.40 (0.28–0.58)
	Pos/Eq (two-tiered)	39/120 (32.5)	63/120 (52.5)	66/120 (55.0)	0/38 (0)	∞	0.45 (0.37–0.55)
CDC	Pos (single-tier)	19/120 (15.8)	46/120 (38.3)	52/120 (43.3)	2/38 (5.3)	8.2 (2.1–32.2)	0.60 (0.50–0.71)
	Pos (two-tiered) ^d^	19/120 (15.8)	31/120 (25.8)	37/120 (30.8)	0/38 (0)	∞	0.69 (0.61–0.78)

FPR, false-positive rate; LR (+), positive likelihood ratio; LR (−), negative likelihood ratio; Pos, proportion positive; Pos/Eq, proportion either positive or equivocal; ∞, infinity; WB, Western blot. ^a^ Both Criteria A and B utilize the 31- and 34-kDa bands, but no equivocal results were observed using Criteria A. ^b^ Samples meeting two-tiered criteria were either positive or equivocal by both EIA and their respective immunoblot criteria. CDC two-tiered criteria utilized only IgG immunoblots for LD diagnosis more than 30 days after disease onset. ^c^ FPR based on results in healthy controls from both endemic and non-endemic areas. ^d^ Fifteen IgM immunoblots positive by single-tier CDC criteria were negative using a two-tiered approach (either EIA-negative or positive only by IgM immunoblot more than 30 days after disease onset). Sensitivity for standard two-tiered CDC criteria was significantly less than either two-tiered Criteria A (30.8% versus 46.7%, respectively, *p* = 0.017 by two-tailed Fisher’s exact test) or two-tiered Criteria B (30.8% versus 55.0%, respectively, *p* = 0.0002 by two-tailed Fisher’s exact test).

**Table 9:** Random effect meta-analysis of single-tier and two-tiered performance of CDC criteria using combined IgG and IgM immunoblots.

	Dataset	Pos (HC) Single-Tier (%)	Pos (HC) Two-Tiered (%)	Pos (HC + XR) Two-Tiered (%)
FPR of individual datasets ^a,b^	MarDx Marblot WB (Trevejo) ^c^	2/38 (5.3)	0/38 (0)	NA
	MarDx Marblot WB (Johnson)	2/113 (1.8)	0/113 (0)	11/224 (4.9)
	Viramed ViraStripe LB (LSR)	22/203 (10.8)	4/203 (2.0)	12/347 (3.5)
Meta-analysis of FPR (95% CI) ^c^	All 3 datasets	6.1% (1.3–13.9)	1.0% (0.1–2.9)	4.2% (2.7–5.9)
Sensitivity of individual datasets for early Lyme disease ^d^	MarDx Marblot WB (Trevejo)	52/120 (43.3)	37/120 (30.8)	NA
	MarDx Marblot WB (Johnson)	42/58 (72.4)	37/58 (63.8)	37/58 (63.8)
	Viramed ViraStripe LB (LSR)	52/78 (66.7)	45/78 (57.7)	45/78 (57.7)
Meta-analysis of Sensitivity (95% CI) ^c^	All 3 datasets	60.5% (41.9–77.6)	50.3% (29.6–70.9)	60.2% (51.9–68.2)
LR (+) ^e^	All 3 datasets	9.9	50.3	14.3
LR (−) ^e^	All 3 datasets	0.42	0.50	0.42

WB; Western blot; LB, line blot; FPR, false-positive rate; Pos, proportion positive; CI, confidence interval; HC, healthy controls; HC + XR, healthy controls plus controls with potentially cross-reacting medical conditions; two-tiered, samples were positive by immunoblot and either positive or equivocal by EIA; LSR, CDC Lyme serum repository; LR (+), positive likelihood ratio; LR (−), negative likelihood ratio; NA, not applicable. ^a^ Healthy controls utilized sera collected from both endemic and non-endemic areas when available (see Supplementary File S2 for additional dataset descriptions). ^b^ Separate analyses of two-tiered specificity were conducted using sera from: (i) healthy controls and (ii) healthy controls plus individuals with potentially cross-reacting medical conditions. ^c^ Controls with potentially cross-reacting conditions were not available for the MarDx Marblot WB (Trevejo [8]) dataset; analyses that included potentially cross-reacting conditions used only 2 datasets. ^d^ For two-tiered CDC criteria, only IgG immunoblots were used for diagnosis of Lyme disease more than 30 days after disease onset. ^e^ Point estimates of likelihood ratios were calculated using composite FPRs and sensitivities for CDC criteria and are reported for heuristic comparison to other criteria.

**Table 10:** Random effect meta-analysis of single-tier and two-tiered performance of Criteria A using combined IgG and IgM immunoblots (without 31- and 34-kDa bands).

Analysis	Dataset	Pos (%) ^a^	Pos Two-Tiered (%) ^b^
FPR for individual datasets ^c^	MarDx Marblot WB (Trevejo)	2/38 (5.3)	0/38 (0)
	Immunetics QualiCode WB	107/429 (24.9) ^d^	NA
	Viramed ViraStripe LB (LSR)	36/203 (17.7)	6/203 (3.0)
Meta-analysis of FPR (95% CI) ^e^	MarDx Marblot WB (Trevejo) and Viramed ViraStripe LB (LSR); (*N* = 241)	12.4% (3.3–26.1)	2.4% (0.5–5.6)
Sensitivity of individual datasets for early Lyme disease	MarDx Marblot WB (Trevejo)	64/120 (53.3)	55/120 (45.8)
	Viramed VirMarblot LB (LSR)	61/78 (78.2)	56/78 (71.8)
Meta-Analysis of Sensitivity (95% CI) ^e^	MarDx Marblot (Trevejo) and Viramed ViraStripe LB (LSR); (*N* = 198)	65.9% (40.6–87.2)	58.8% (33.3–82.0)
LR (+) ^f^ LR (−) ^f^	MarDx Marblot WB (Trevejo) and Viramed ViraStripe LB (LSR)	5.3 0.39	24.5 0.42

See legend from Table 9 for abbreviations. ^a^ After omitting the 31- and 34-kDa bands from Criteria A, there were no equivocal results observed. ^b^ Two-tiered test results were not available for the Immunetics QualiCode WB database due to lack of concurrent EIA data. ^c^ FPR utilized healthy controls from both endemic and non-endemic areas. ^d^ Individual IgM band data were not available for the Immunetics QualiCode WB database, so only IgG immunoblot data are reported. ^e^ The Immunetics QualiCode WB dataset was excluded from this meta-analysis because of missing IgM and EIA data. ^f^ Point estimates of likelihood ratios were calculated using composite FPRs and sensitivities for Criteria A and are reported for heuristic comparisons to other criteria.

**Table 11:** Random effect meta-analysis of single-tier and two-tiered performance of Criteria B using combined IgG and IgM immunoblots (without 31- and 34-kDa bands).

Analysis	Dataset	Pos (%)	Pos/Eq (%)	Pos/Eq Two-Tiered (%) ^a^
FPR for individual datasets ^b,c^	MarDx Marblot WB (Trevejo)	1/38 (2.6)	11/38 (26.3)	0/38 (0)
	Immunetics QualiCode WB	2/429 (0.5)	109/429 (25.4)	NA
	Viramed ViraStripe LB (LSR)	18/203 (8.9)	73/203 (35.9)	10/203 (4.9)
Meta-analysis of FPR (95% CI) ^d^	MarDx Marblot WB (Trevejo) and Viramed ViraStripe LB (LSR); (*N* = 241)	7.3% (3.0–13.3)	35.0% (29.1–41.1)	2.9% (0.09–9.5)
Sensitivity of individual datasets for early Lyme disease	MarDx Marblot WB (Trevejo)	48/120 (40.0)	87/120 (72.5)	66/120 (55.0)
	Viramed VirMarblot LB (LSR)	48/78 (61.5)	65/78 (83.3)	59/78 (75.6)
Meta-Analysis of Sensitivity (95% CI) ^d^	MarDx Marblot (Trevejo) and Viramed ViraStripe LB (LSR); (*N* = 198)	50.5% (30.0–70.9)	77.5% (66.3–87.0)	65.3% (44.4–83.5)
LR (+) ^e^ LR (−) ^e^	MarDx Marblot WB (Trevejo) and Viramed ViraStripe LB (LSR)	6.9 0.53	2.2 0.35	22.5 0.36

Two-tiered, samples meeting two-tiered criteria were either positive or equivocal by both immunoblot and EIA methods; Pos, proportion positive; Pos/Eq, proportion either positive or equivocal. See legend from Table 9 for additional abbreviations. ^a^ Two-tiered test results were not available for the Immunetics QualiCode WB database due to lack of concurrent EIA data. ^b^ FPR utilized healthy controls from both endemic and non-endemic areas. ^c^ Individual IgM band data were not available for the Immunetics QualiCode WB database, so only IgG immunoblot data are reported. ^d^ The Immunetics QualiCode WB dataset was excluded from this meta-analysis because of missing IgM and EIA data. ^e^ Point estimates of likelihood ratios were calculated using composite FPRs and sensitivities for Criteria B and are reported for heuristic comparisons to other criteria.

**Table 12:** Comparative performance of two-tiered CDC criteria using immunoblots, MTT (all-EIA) criteria, and alternative immunoblot criteria using the CDC LSR ^a^.

Criteria	Sensitivity ^b^ (% with 95% CI)	FPR in Healthy Controls (% with 95% CI)	Total FPR ^c^ (% with 95% CI)	LR (+) (95% CI)	LR (−) (95% CI)	Pretest Prob ^d^ (% with 95% CI)
Criteria A (Pos) ^e^	61/78 (78.2; 68.8–87.6)	36/203 (17.7; 12.4–23.0)	64/347 (18.4; 14.3–22.5)	4.2 (3.3–5.4)	0.27 (0.18–0.41)	19.2 (15.6–23.2)
Criteria A (Pos) two-tiered ^e,f^	56/78 (71.8; 61.6–82.0)	6/203 (3.0; 0.6–5.3)	19/347 (5.5; 3.1–7.9)	13.1 (8.3–20.7)	0.30 (0.21–0.43)	7.1 (4.6–10.8)
Criteria B (Pos)	48/78 (61.5; 50.5–72.6)	18/203 (8.9; 4.9–12.8)	33/347 (9.5; 6.4–12.6)	6.5 (4.5–9.4)	0.43 (0.32–0.56)	13.3 (9.6–18.2)
Criteria B (Pos) two-tiered ^f^	46/78 (59.0; 47.8–70.1)	3/203 (1.5; 0–3.2)	12/347 (3.5: 1.5–5.4)	17.1 (9.5–30.6)	0.43 (0.33–0.56)	5.5 (3.2–9.5)
Criteria B (Pos/Eq)	65/78 (83.3; 74.9–91.8)	73/203 (36.0; 29.3–42.6)	125/347 (36.0; 31.0–41.1)	2.3 (1.9–2.7)	0.26 (0.16–0.43)	30.0 (27.0–34.5)
Criteria B (Pos/Eq) two-tiered ^f^	59/78 (75.6; 65.9–85.4)	10/203 (4.9; 1.9–7.9)	30/347 (8.6; 5.7–11.6)	8.7 (6.1–12.6)	0.27 (0.18–0.40)	10.3 (7.4–14.1)
Standard CDC two-tiered criteria ^f^	45/78 (57.7; 46.5–68.9)	4/203 (2.0; 0–3.9)	12/347 (3.5; 1.5–5.4)	16.7 (9.3–30.0)	0.44 (0.34–0.57)	5.6 (3.2–9.7)
Modified two-tiered VIDAS/C6 EIA ^g^	52/78 (66.7; 56.0–77.4)	1/203 (0.5; 0–1.5)	5/347 (1.4; 0.2–2.7)	46.3 (19.1–112.0)	0.34 (0.25–0.46)	1.8 (0.9–4.9)
Modified two-tiered VlsE1/pepC10 EIA ^h^	50/60 (83.3; 72.0–90.7)	0/100 (0)	2/190 (1.1; 0.3–3.8)	79.2 (19.9–315.7)	0.17 (0.10–0.30)	1.2 (0.3–4.8)

Two-tiered, samples meeting two-tiered criteria were either positive or equivocal by both EIA and their respective immunoblot criteria; Pretest prob., minimum pretest probability of Lyme disease required for a test positive by a given criterion to demonstrate a PPV ≥ 50% for that criterion. See legend in Table 9 for additional abbreviations. ^a^ Data from Molins et al. [5] for combined IgG and IgM antibodies to *B. burgdorferi* by immunoblot. Data from Sfeir et al. [3] for modified two-tiered VlsE1/pepC10 EIA results. The 31- and 34-kDa IgG and IgM band results were not recorded for this dataset. ^b^ Sensitivity included either IgG or IgM antibodies among patients with early Lyme disease (≤60 days after disease onset). ^c^ Total FPR included either IgG or IgM antibodies in 203 healthy control sera (from both endemic and non-endemic areas) plus 144 sera from patients with potentially cross-reacting medical conditions. ^d^ This value represents the minimum pretest probability of Lyme disease necessary for a test positive by a given criterion or assay to be correct at least 50% of the time (i.e., positive predictive value ≥ 50%). ^e^ No equivocal results were observed using Criteria A. ^f^ The same alternative immunoblot interpretive criteria, whether single-tier or two-tiered, were applied to early LD and control sera, regardless of disease duration. Standard two-tiered CDC criteria utilize only IgG immunoblots for diagnosis more than 30 days after disease onset; the latter criteria were also applied to control samples when the duration of illness was known, leading to categorizing 2 controls with rheumatoid arthritis as negative by CDC criteria (see Supplementary File S2). ^g^ This modified two-tiered (MTT) approach utilizes a first-tier VIDAS EIA for polyvalent IgG/IgM antibodies to *B. burgdorferi,* followed by confirmation of positive or equivocal first-tier results by an EIA for IgG/IgM antibodies to C6 peptide [5]. ^h^ Samples positive or equivocal by the Zeus ELISA Borrelia VlsE1/pepC10 IgG/IgM Test System, a polyvalent EIA using both antigens, are confirmed by second-tier monovalent IgG and IgM whole-cell EIAs; samples that are either positive or equivocal for either IgG or IgM antibodies by whole-cell EIA to *B. burgdorferi* are considered MTT positive [3].

## Data Availability

Except for the CDC Lyme Serum Repository [5,69], all data used for this article are included in the text and Supplementary Materials. Interested individuals may contact the CDC, Division of Vector-Borne Diseases, Fort Collins, CO, USA to inquire about data from the CDC Lyme Serum Repository.

## Supplementary Materials

The following are available online at https://www.mdpi.com/article/10.3390/pathogens12111282/s1, Table S1. IgG and IgM immunoblot band frequencies among healthy endemic controls; Table S2. IgG and IgM immunoblot band frequencies among healthy non-endemic controls; Table S3. (A) IgG and IgM band frequencies among cross-reacting controls, and (B) IgG immunoblot band cutoff criteria; Supplementary File S1. Alternative immunoblot criteria from Laboratory B; Supplementary File S2. Description of datasets 1–8; Supplementary File S3. Immunetics QualiCode *B. burgdorferi* IgG Western blot controls (510(k), dataset 1); Supplementary File S4. MarDx Marblot IgG and IgM Western blots (Trevejo et al. [8], dataset 4).
