# Supplementary material for: Immunoblot Criteria for Diagnosis of Lyme Disease: A Comparison of CDC Criteria to Alternative Interpretive Approaches
Source: Pathogens. 2023 Oct 26;12(11):1282. doi: 10.3390/pathogens12111282 (PMC10674374; doi:10.3390/pathogens12111282)
Supplement: Supplementary file 1 [file pathogens-12-01282-s001.zip › pathogens-2456134_Supplementary_File_S2.pdf]

pathogens

MDPI

Article

Immunoblot Criteria for Diagnosis of Lyme Disease: A Comparison of CDC Criteria to Alternative Interpretive Approaches

Richard Porwancher [1,2,*], Andrew Levin [3] and Rosalie Trevejo [4]

[1] Section of Allergy, Immunology, and Infectious Diseases, Department of Medicine, Rutgers Robert Wood Johnson Medical School, New Brunswick, NJ 08901, USA
[2] Princeton Infectious Diseases Associates, LLC, Plainsboro, NJ 08536, USA
[3] Kephera Diagnostics, LLC, Framingham, MA 01702, USA; alevin@kephera.com
[4] Epidemiologist, Acute and Communicable Disease Prevention, Oregon Health Authority, Portland, OR 97232, USA; Rosalie.Trevejo2@oha.oregon.gov
* Correspondence: porwancher@aol.com

Supplementary File S2. Description of Datasets 1-8

1. Immunetics QualiCode *B. burgdorferi* IgG and IgM Western blot kits (510(k) applications K991063 and K991062, respectively, Immunetics, Inc., Cambridge, MA, USA).

IgG Western blot band results were recorded on individual specimens, including the 31- and 34-kDa bands. Western blots were visually read and band locations were identified using monoclonal antibodies obtained from the CDC Division of Vector-Borne Diseases (Fort Collins, CO, USA). Specimens included 278 control sera from healthy patients residing in endemic areas and 151 control sera from healthy patients residing in non-endemic areas; all samples were tested and interpreted using CDC-advocated methods [1], permitting the comparison of false-positive rates (FPRs) of CDC interpretative criteria to alternative Criteria A and B in healthy controls for IgG Western blots. Individual IgG Western blot results are listed in Supplementary File F3. Only summary immunoblot data were available for IgM Western blots from the Immunetics QualiCode 510(k) application K991062; the latter immunoblots did not include data concerning IgM antibodies to the 31- and 34-kDa bands. Data from these two 510(k) applications included: (i) summary information concerning IgG Western blot sensitivity for CDC criteria for 107 early LD sera collected within 60 days of disease onset, as well as band frequency in healthy endemic and non-endemic controls (Supplementary Tables S1 and S2); (ii) summary information concerning IgM Western blot sensitivity for CDC criteria for 99 early LD sera collected within 60 days of disease onset, as well as band frequency in healthy endemic and non-endemic controls (Supplementary Tables S1 and S2); (iii) summary information concerning the specificity of CDC IgG Western blot criteria in 172 sera from patients with potentially cross-reacting conditions (i.e., anti-nuclear antibody, *Helicobacter pylori* infection, HIV infection, mononucleosis, periodontal disease, rheumatoid arthritis, tick-borne relapsing fever, systemic lupus erythematosus, syphilis, and tularemia antibodies). No concurrent EIA data were available. Individual 510(k) application summaries are available through: https://www.accessdata.fda.gov/scripts/cdrh/cfdocs/cfPMN/pmn.cfm (accessed on 2 January 2023).

2. EUROIMMUN Anti-*Borrelia burgdorferi* US IgG and IgM Western blot kits (510(k) applications K161513 and K172722, respectively, EUROIMMUN US, Mountain Lakes, NJ).

Summary information for IgG and IgM Western blot performance was available from the EUROIMMUN 510(k) applications for sera from 34 patients with early LD (less than 90 days after disease onset), 98 control sera from healthy patients residing in LD-endemic communities, and 100 control sera from healthy patients residing in non-endemic

communities. Immunoblots were tested and interpreted using CDC-advocated methods [1]. No information was available concerning the 31- and 34-kDa bands and no concurrent EIA data were available. Visually-read immunoblot results were used for this dataset. Immunoblot band results for individual samples were not available. Summary information about band frequencies in IgG and IgM immunoblots in healthy controls is reported in Supplementary Tables S1 and S2.

3. MarDx *B. burgdorferi* Marblot Strip Test System (separate IgG and IgM Western blot kits, 510(k) applications K950279 and K951709, respectively, MarDx Diagnostics, Inc., Carlsbad, CA, USA).

Summary information for IgG and IgM Western blot performance was available from the MarDx Marblot 510(k) applications for sera from 273 patients with early LD (less than 60 days after disease onset) and 514 control sera from healthy patients residing in LD-endemic and non-endemic communities (combined). Immunoblot band results on individual samples were not available. Samples were tested and interpreted using CDC-advocated methods [1]. Immunoblots were interpreted visually. No information was available concerning the 31- and 34-kDa bands and no concurrent EIA data were available. Summary information on band frequencies in IgG and IgM immunoblots in healthy controls is reported in Supplementary Table S1.

4. MarDx *B. burgdorferi* Marblot Strip Test System (separate IgG and IgM Western blot kits, Trevejo et al. [8]).

IgG and IgM Western blot band data from individual specimens tested using MarDx Marblot test kits (MarDx Diagnostics, Inc., Carlsbad, CA, USA), including the 31- and 34-kDa bands, were available from a study by Trevejo et al. [8], sponsored by the CDC, Division of Vector-Borne Diseases, Fort Collins, CO. Western blots were visually read and band locations were identified using monoclonal antibodies. There were minor differences between the data published by Trevejo et al. [8] and the dataset used for this paper, partly due to loss of sera from repeat testing. Sixty-seven early-acute-phase sera were collected a median of 4 days after developing erythema migrans (range 0 to 19 days). Fifty-five early-convalescent-phase sera were collected a median of 36 days after disease onset (range 21 to 161 days), but immunoblots were available for only 53 convalescent sera. A total of 38 control sera were collected from healthy blood donors residing in LD-endemic communities. No sera from patients with potentially cross-reacting medical conditions were studied. Both IgG and IgM immunoblots as well as polyvalent IgG/IgM EIAs (IgG/IgM VIDAS EIA, bioMérieux Vitek, Hazelwood, MO, USA) were performed on all samples. Immunoblots were interpreted using CDC criteria [1], Criteria A, and Criteria B; test results were compared among patients with early disease and among healthy endemic controls. Individual IgG and IgM immunoblot band frequencies in healthy endemic controls are reported in Supplementary Table S1 and band results for individual specimens are reported in Supplementary File S4. A total of 15 early-convalescent-phase sera, positive by IgM Western blot but negative by IgG Western blot using CDC criteria, were considered two-tier negative because the samples were either EIA-negative or were collected more than 30 days after disease onset. Although the latter samples were reported by Trevejo et al. [8], our dataset did not specifically identify those samples collected more than 30 days after disease onset.

5. MarDx *B. burgdorferi* Marblot Strip Test System (separate IgG and IgM Western blot kits, Johnson et al. [26]).

A flagellin-based EIA and IgG and IgM Western blots were performed on all samples using the MarDx Marblot test kits (MarDx Diagnostics, Inc., Carlsbad, CA, USA) and interpreted using CDC criteria [1]. Samples included sera from 58 patients with early-acute-phase LD (erythema migrans), 113 control sera from healthy patients residing in non-endemic communities, and 111 control sera from patients with potentially cross-reacting medical conditions, including autoimmune disorders, leptospirosis, periodontitis,

relapsing fever, syphilis, tularemia, and other conditions. Antibodies to the 31- and 34-kDa bands were not recorded and immunoblot band results on individual specimens were not available. Combined IgG and IgM immunoblot results were available, but separate IgG and IgM results were not reported.

6. Trinity Biotech Lyme *B. burgdorferi* MarStripe Tests (separate IgG and IgM line blots, 510(k) applications K163095 and K172254, respectively, Trinity Biotech USA, Jamestown, NY).

The 10 CDC-advocated antigens were produced in recombinant form and employed as IgG and IgM line blots. OspA and OspB antigens were not utilized. Line blots were performed on all samples and interpreted using CDC criteria [1]. No concurrent EIA data were available and immunoblot band results on individual specimens were not reported. Summary information was available for IgG and IgM line blot results, including 22 sera from patients with Stage 1 early LD (within 4 weeks of disease onset) for IgG line blot testing, 19 sera from patients with stage 1 early LD (within 4 weeks of disease onset) for IgM line blot testing, and 220 control sera from healthy patients residing in endemic and non-endemic communities (combined panel). Summary IgG and IgM band frequencies in healthy controls are reported in Supplementary Table S1.

7. Gold Standard Diagnostics *Borrelia burgdorferi* B31 IgG and IgM Line Blot Test Kits (510(k) applications K113847 and K113846, respectively, Gold Standard Diagnostics, Budapest, Hungary).

The 10 CDC-advocated antigens were produced in either recombinant form or through purification methods and then employed as IgG and IgM line blots. OspA and OspB antigens were not utilized. Immunoblots were performed on all samples and interpreted using CDC criteria [1], Criteria A, and Criteria B; test results were compared among patients with early disease and among healthy endemic controls. No concurrent EIA data were available and immunoblot band results on individual specimens were not reported. Summary information was available for IgG and IgM line blot results, including 40 sera from patients with early LD collected within 60 days of disease onset, 119 control sera from healthy patients residing in non-endemic communities, and 115 control sera from healthy patients residing in endemic communities. Summary information concerning band frequencies in healthy controls is listed in Supplementary Tables S1 and S2.

8. Viramed Biotech AG *Borrelia* B31 ViraStripe IgG and IgM line blots and MarDx *B. burgdorferi* Marblot Strip Test System (separate IgG and IgM Western blot kits), Molins et al. [5,69].

Partially-blinded immunoblot and EIA data from studies performed by Molins et al. [5,69] were obtained under a materials transfer agreement between one co-author (R.P.) and the Centers for Disease Control and Prevention (Fort Collins, CO, USA). The latter studies were performed using the CDC's Lyme Serum Repository (LSR), a prospective collection of sera available to researchers and medical device manufacturers to assist in development of LD diagnostic assays [69]; specimens are provided by the CDC for testing in a blinded manner. The VIDAS Lyme IgG and IgM polyvalent whole-cell EIA (LYT) (bioMérieux, Inc., Durham, NC, USA) and an EIA for polyvalent IgG and IgM antibodies to the C6 peptide (Immunetics, Inc., Cambridge, MA, USA) were performed on all samples. Viramed Biotech AG *Borrelia* B31 ViraStripe IgG and IgM line blots (Viramed Biotech AG, Germany) and MarDx *B. burgdorferi* Marblot Strip Test System IgG and IgM Western blots (MarDx Diagnostics, Inc., Carlsbad, CA, USA) were performed on all samples, regardless of EIA results. Individual sample immunoblot band results were available for Viramed ViraStripe IgG and IgM line blots, but only summary data were available for the MarDx Marblot IgG and IgM Western blots; all immunoblots were interpreted using CDC criteria [1], Criteria A, and Criteria B. The Viramed ViraStripe line blot kits utilize antigens purified by electrophoretic and chromatographic techniques for both IgG and IgM line blots. Specimens included sera from 40 patients with early-acute-phase disease collected

within 4 weeks of disease onset, 38 sera from early-convalescent-phase disease collected within 45 days of disease onset, 7 patients with Lyme carditis, 10 patients with early Lyme neuroborreliosis, and 29 patients with late disease (Lyme arthritis). Based on analyses of the data provided directly by the CDC and prior publications by Molins et al. [5,69], four early-convalescent-phase sera were positive only by EIA and IgM immunoblot but fell beyond the 30-day cutoff for diagnostic use of IgM immunoblotting; these specific specimens were not individually identified by the CDC, but summary data concerning two-tiered performance were available for all sample categories from Molins' studies [5,69]. The lack of information concerning which IgM immunoblots failed to meet this 30-day cutoff did not affect application of CDC interpretive criteria for single-tier use of IgM and IgG immunoblots, or application of alternative interpretive Criteria A or B for the diagnosis of either early or late LD. Sera from 144 patients with potentially cross-reacting medical conditions, 101 control sera from healthy individuals living in non-endemic communities, and 102 control sera from healthy individuals living in endemic communities were available for testing. Potentially cross-reacting medical conditions included fibromyalgia, severe periodontitis, rheumatoid arthritis, syphilis, multiple sclerosis, and infectious mononucleosis. Two samples from patients with rheumatoid arthritis were positive only by EIA and IgM immunoblot more than 30 days after disease onset, therefore failing to meet the CDC's two-tiered diagnostic criteria; the identities of these specific specimens were not provided by the CDC, but summary data concerning the performance of CDC's two-tiered diagnostic criteria among controls were available for all sample categories from Molins' studies [5,69]. Individual IgG and IgM immunoblot band results were available for all specimens, but did not include OspA and OspB antigens (i.e., 31- and 34-kDa bands). The specificity of alternative interpretive Criteria A and B in both healthy controls and patients with potentially cross-reacting conditions are reported, although without the latter two bands. Visually-read immunoblot results were used for this dataset. Summary immunoblot band frequencies for both the Viramed ViraStripe line blot and MarDx Marblot Western blot in healthy endemic, healthy non-endemic, and potentially cross-reacting control sera are listed in Supplementary Tables S1, S2, and S3 (section A), respectively.

A total of 30 early-acute-phase sera from patients with early LD, 30 early-convalescent-phase sera, 100 healthy control sera, and 90 sera from patients with potentially cross-reacting conditions from the LSR were utilized by Sfeir et al. [3] for validation of a new FDA-cleared modified two-tiered approach to serodiagnosis (Zeus ELISA VlsE1/pepC10 IgG/IgM Test System, Zeus Scientific, Inc., Branchburg, NJ, USA); these samples were chosen in advance from the LSR by the CDC as a standard for pre-marketing assessment prior to FDA clearance [69]. All samples from patients with early LD were collected within 60 days of disease onset. A first-tier EIA for polyvalent IgG and IgM antibodies to recombinant VlsE1 from the B31 strain of *B. burgdorferi* and pepC10 antigen was performed; either positive or equivocal first-tier samples were confirmed using two monovalent whole-cell EIAs (Zeus Scientific, Inc., Branchburg, NJ, USA), one for IgG antibodies, and the other for IgM antibodies to the B31 strain of *B. burgdorferi*. A positive or equivocal result to either second-tier EIA was necessary to consider the overall MTT assay positive. The performance of the MTT using the Zeus ELISA VlsE1/pepC10 IgG/IgM Test System is reported in Table 12. Although results for alternative Criteria A and B were not available for this validation subset, results for alternative criteria are reported using the entire LSR; it is assumed that this validation subset constitutes a representative sample of the overall LSR dataset. A second MTT assay was performed using the entire Lyme Serum Repository: samples positive or equivocal by a first-tier VIDAS EIA for polyvalent IgG/IgM antibodies to *B. burgdorferi* were confirmed using the Immunetics C6 EIA for polyvalent IgG/IgM antibodies [5]. Positive or equivocal results for both tiers were required to consider the overall MTT assay positive.

Disclaimer/Publisher's Note: The statements, opinions and data contained in all publications are solely those of the individual author(s) and contributor(s) and not of MDPI and/or the editor(s). MDPI and/or the editor(s) disclaim responsibility for any injury to people or property resulting from any ideas, methods, instructions or products referred to in the content.
