# Supplementary material for: Immunoblot Criteria for Diagnosis of Lyme Disease: A Comparison of CDC Criteria to Alternative Interpretive Approaches
Source: Pathogens. 2023 Oct 26;12(11):1282. doi: 10.3390/pathogens12111282 (PMC10674374; doi:10.3390/pathogens12111282)
Supplement: Supplementary file 1 [file pathogens-12-01282-s001.zip › Supplementary File S1. Alternative immunoblot criteria from Laboratory B.pdf]

MEDICAL DIAGNOSTIC LABORATORIES, L.L.C.

INTERPRETATION GUIDELINES

WESTERN BLOTS, C6 ELISAS, LYME IgG / IgM ELISAS

Lyme IgG and IgM Western blot Kits - This kit is a membrane immunoassay based on the Western blot method. As recommended by the CDC, all samples which test positive or indeterminate on a serological screening test should be re-tested on a *B. burgdorferi* Western blot test. *B. burgdorferi* Western blot IgG and IgM assays are recommended **for the evaluation of sera from patients believed to be in the first four weeks of infection, while an IgG assay alone is** recommended for evaluation of sera from patients with symptoms of late Lyme disease.

Report Key - The Alternative Interpretive Criteria is based upon a study published by Dr. Richard Tilton (Tilton RC, Sand MN, Manak M. (1997). The Western Immunoblot for Lyme Disease: Determination of Sensitivity, Specificity, and Interpretive Criteria with Use of Commercially Available Performance Panels. Clinical Infectious Disease. 25:S31-4). Reprints are available upon request.

***Effective for results verified on or after 10/17/2013**. To be considered positive, bands must have a value of 60 or greater.

	Result	CDC Criteria (Antibody.CDC)	Alternative Criteria (Antibody.Alt)
IgM	Negative (Non-reactive)	Fewer than 2 bands: 23, 39, 41	**No Lyme specific bands**
	Negative (Equivocal)	--	One band must be present: 23, 31, 34, 39, 41
	Positive (Reactive)	Two or more bands must be present: 23, 39, 41	Two or more bands must be present: 23, 31, 34, 39, 41
IgG	Negative (Non-reactive)	Fewer than 5 bands: 18, 23, 28, 30, 39, 41, 45, 58, 66, 93	**No Lyme specific bands**
	Negative (Equivocal)	--	One or two bands must be present: 23, 31, 34, 39, 93
	Positive (Reactive)	Five or more bands must be present: 18, 23, 28, 30, 39, 41, 45, 58, 66, 93	Three or more bands must be present: 23, 31, 34, 39, 93

Negative Results: Additional specimens should be submitted in 2-4 weeks if *B. burgdorferi* exposure has not been ruled out.

Positive Results: The corresponding antibodies (IgG or IgM) to significant *B. burgdorferi* proteins detected; presumptive evidence of probable exposure.

The use of the Lyme Western blot on Cerebrospinal fluid is an off-label application and is for investigational *use only. This test kit was designed for human serum specimens. There is no validation data available for* ***the use of this test on human Cerebrospinal fluid (CSF) specimens.***

The major differences between the interpretative criteria are:

1. **IgM – The Alternative Criteria is similar to the CDC except that the 93 band has been included as a significant IgM band specific to** *B. burgdorferi*.
2. **IgG – The Alternative Criteria is based on both the number of bands and the significance of the antibodies** detected. For example, Osp A (31) and Osp B (34) are important bands seen often in late stages of Lyme disease.
3. **There is an "equivocal" category for both IgG and IgM blots which indicates that although there are not sufficient antibody bands present for the blot to be reactive, there is significant immunologic activity that may be related** to Lyme disease.

Toll Free: (877) 269-0090 • Fax: (609) 570-1050 • www.mdlab.com
A MEMBER OF GENESIS BIOTECHNOLOGY GROUP

View: MF	Lyme
Mail: Yes One Yes	Fax: Yes One Manual No

Medical Director, Mats Sanden, M.D.

PATH

11/14/2019
Final
