# Supplementary material for: Immunoblot Criteria for Diagnosis of Lyme Disease: A Comparison of CDC Criteria to Alternative Interpretive Approaches
Source: Pathogens. 2023 Oct 26;12(11):1282. doi: 10.3390/pathogens12111282 (PMC10674374; doi:10.3390/pathogens12111282)
Supplement: Supplementary file 1 [file pathogens-12-01282-s001.zip › Supplementary Table S1. IgG and IgM immunoblot band frequencies among healthy endemic controls.pdf]

Healthy endemic control sera datasets (#subjects)	Band Frequency												
IgG immunoblot band kDa (B31 strain)	93(a)	66	58	45	41	39	34	31	30	28	23(b)	18	IgG FP Rate (%)
Immunetics QualiCode Western blot 510(k) (278)	0.13	0.24	0.1	0.07	0.93	0.13	0.1	0.12	0.11	0.01	0.11	0.1	0.7
EUROIMMUN Western blot 510(k) (98)	0.1	0.14	0.14	0.06	0.84	0.04	NA	NA	0.07	0.01	0.03	0.1	3.1
MarDx Marblot Western blot 510(k) (291)*	0.03	0.04	0.12	0.01	0.8	0.27	NA	NA	0.01	0	0.04	0	1*
MarDx Marblot Western blot (Trevejo) (38)	0.05	0.08	0.03	0.05	0.66	0.05	0	0	0	0.03	0	0.05	2.6
MarDx Marblot Western blot (LSR) (101)	0.01	0.08	0.08	0.03	0.37	0.03	NA	NA	0.02	0	0.09	0.04	2
Trinity Biotech MarStripe line blot 510(k) (219)*	0.02	0.04	0.01	0	0.06	0.18	NA	NA	0.01	0.01	0.03	0.01	0.5*
Viramed ViraStripe line blot (LSR) (101)	0.1	0.39	0.18	0	0.54	0.05	NA	NA	0.03	0.02	0.15	0.1	4
Gold Standard Diagnostics Line blot 510(k) (119)	0.04	0.01	0.06	0.04	0.33	0.06	NA	NA	0.02	0.01	0.05	0.05	1.7
IgM immunoblot band kDa (B31 strain)	93(a)	41	39	34	31	23(b)							IgM FP Rate (%)
Immunetics QualiCode Western blot 510(k) (279)	NA	0.3	0.03	NA	NA	0.06							4.7
EUROIMMUN Western blot 510(k) (98)	NA	0.84	0.04	NA	NA	0.03							5.1
MarDx Marblot Western blot 510(k) (291)*	NA	0.21	0.03	NA	NA	0.09							5*
MarDx Marblot Western blot (Trevejo) (38)	0.05	0.05	0.08	0	0.03	0.11							2.6
MarDx Marblot Western blot (LSR) (101)	NA	0.05	0	NA	NA	0.23							2
Trinity Biotech MarStripe line blot 510(k) (220)*	NA	0.01	0.01	NA	NA	0.02							0.5*
Viramed ViraStripe line blot LSR (101)	NA	0.17	0.2	NA	NA	0.08							11.9
Gold Standard Diagnostics line blot 510(k) (119)	NA	0.01	0.02	NA	NA	0.04							0

Legend: FP, false-positive; LSR, CDC Lyme Serum Repository samples; kDa, kilodaltons; 510(k), medical device application to FDA
(a) Reports 83- to 100-kDa molecular weight band
(b) Reports 21- to 25-kDa molecular weight band (OspC)
*includes both endemic and non-endemic controls

NOTE: See text and Supplementary File S2 for dataset descriptions
