# Supplementary material for: Immunoblot Criteria for Diagnosis of Lyme Disease: A Comparison of CDC Criteria to Alternative Interpretive Approaches
Source: Pathogens. 2023 Oct 26;12(11):1282. doi: 10.3390/pathogens12111282 (PMC10674374; doi:10.3390/pathogens12111282)
Supplement: Supplementary file 1 [file pathogens-12-01282-s001.zip › Supplementary Table S2. IgG and IgM immunoblot band frequencies among healthy non-endemic controls.pdf]

Healthy non-endemic control sera datasets (#subjects)	Band Frequency												FP Rate (%)
IgG immunoblot band kDa (B31 strain)	93(a)	66	58	45	41	39	34	31	30	28	23(b)	18	FP Rate (%)
Immunetics QualiCode Western blot 510(k) (151)	0.1	0.17	0.07	0.05	0.93	0.07	0.13	0.21	0.16	0.05	0.14	0.05	4
EUROIMMUN Western blot 510(k) (100)	0.08	0.16	0.05	0.04	0.87	0.02	NA	NA	0.13	0	0.02	0.09	0
MarDx Marblot Western blot (LSR) (102)	0	0.02	0.04	0.04	0.29	0.02	NA	NA	0	0.01	0.01	0.01	0
Viramed ViraStripe line blot (LSR) (102)	0.06	0.28	0.07	0.1	0.66	0.04	NA	NA	0.03	0.04	0.28	0.15	1
Gold Standard Diagnostics line blot 510(k) (115)	0.03	0.03	0.04	0.03	0.25	0.04	NA	NA	0.01	0.01	0.02	0	0
IgM immunoblot band kDa (B31 strain)	41	39	23(b)										
Immunetics QualiCode Western blot 510(k) (151)	0.21	0.01	0.05										4
EUROIMMUN Western blot 510(k) (98)	0.87	0.02	0.02										4
MarDx Marblot Western blot (LSR) (102)	0.1	0.03	0.2										2
Viramed ViraStripe line blot (LSR) (102)	0.28	0.23	0.25										3.9
Gold Standard Diagnostics line blot 510(k) (115)	0.03	0.01	0.07										0

Legend: FP, false-positive; LSR, CDC Lyme Serum Repository; kDa, kilodalton; 510(k), medical device application to FDA
(a) reports 83- to 100-kDa molecular weight band
(b) reports 21- to 25-kDa molecular weight band (OspC)

Note: see text and Supplementary File S2 for dataset descriptions
