# Supplementary material for: Immunoblot Criteria for Diagnosis of Lyme Disease: A Comparison of CDC Criteria to Alternative Interpretive Approaches
Source: Pathogens. 2023 Oct 26;12(11):1282. doi: 10.3390/pathogens12111282 (PMC10674374; doi:10.3390/pathogens12111282)
Supplement: Supplementary file 1 [file pathogens-12-01282-s001.zip › Supplementary Table S3. (A) IgG and IgM immunoblot band frequencies among cross-reacting controls, (B) IgG immunoblot band cutoff criteria.pdf]

A. Immunoblot band frequencies in cross-reacting sera from CDC Lyme Serum Repository (Molins et al. [5]) (#subjects)

IgG immunoblot band kDa (B31 strain)	93(a)	66	58	45	41	39	34	31	30	28	23(b)	18	FP Rate (%)
MarDx Marblot Western blot (LSR) (144)	0.01	0.06	0.03	0.01	0.32	0.03	NA	NA	0.01	0	0.01	0.01	0
Viramed ViraStripe line blot (LSR) (144)	0.06	0.19	0.19	0.07	0.58	0.01	NA	NA	0.03	0.02	0.12	0.07	1.4

IgM immunoblot band kDa (B31 strain)	41	39	23(b)	FP Rate (%)
MarDx Marblot Western blot (LSR) (144)	0.08	0.06	0.15	7.6
Viramed ViraStripe line blot (LSR) (144)	0.22	0.21	0.16	10.4

Legend: FP, false-positive; LSR, CDC Lyme Serum Repository; kDa, kilodalton
(a) Reports 83- to 100-kDa molecular weight band
(b) Reports 21- to 25-kDa molecular weight band (OspC)
NOTE: see text for references and both text and Supplementary File S2 for dataset descriptions

B. False-positive rates of single-tier IgG immunoblots in different datasets generated by varying the minimum number of CDC-advocated IgG bands required to consider test positive (# subjects studied)	≥2 bands	≥3 bands	≥4 bands	≥5 bands
Viramed ViraStripe IgG line blot (LSR): includes cross-reacting controls plus healthy endemic and non-endemic controls using CDC-advocated bands (347)	33.70%	14.70%	5.20%	2.30%
Immunetics Qualicode IgG Western blot 510(k): includes healthy endemic and non-endemic controls using CDC-advocated bands (without 31- and 34-kDa bands) (429)	54.50%	24.20%	8.40%	1.90%
Immunetics Qualicode IgG Western blot 510(k): includes healthy endemic and non-endemic controls using CDC-advocated bands plus 31- and 34-kDa bands (429)	59.90%	32.90%	13.50%	5.80%
MarDx Marblot IgG Western blot (Trevejo [8]): includes healthly endemic controls using CDC-advocated bands (38)	13.20%	5.30%	2.60%	2.60%

Legend: LSR, CDC Lyme Serum Repository; 510(k), medical device application to FDA; kDa, kilodalton
NOTE: see text for references and both text and Supplementary File S2 for dataset descriptions
